# The largest theropod track site in Yunnan, China: a footprint assemblage from the Lower Jurassic Fengjiahe Formation

**DOI:** 10.7717/peerj.11788

**Published:** 2021-10-05

**Authors:** Hongqing Li, Claire Peyre de Fabrègues, Shundong Bi, Yi Wang, Xing Xu

**Affiliations:** 1 Centre for Vertebrate Evolutionary Biology, Yunnan University, Kunming, Yunnan Province, China; 2 Department of Biology, Indiana University of Pennsylvania, Indiana, PA, United States of America; 3 Yuxi Museum, Yuxi, Yunnan Province, China; 4 Key Laboratory of Vertebrate Evolution and Human Origins, Institute of Vertebrate Paleontology and Paleoanthropology, Chinese Academy of Sciences, Beijing, China; 5 Center for Excellence in Life and Paleoenvironment, Chinese Academy of Sciences, Beijing, China

**Keywords:** Theropoda, Grallator, Kayentapus, Jurassic, Lower Jurassic, Fengjiahe, Yunnan, China, Ichnotaxonomy, Tracks

## Abstract

Yunnan Province is famous for its diversified Lufeng vertebrate faunas containing many saurischian dinosaur remains. In addition to the body fossil record, dinosaur ichnofossils have also been discovered in Yunnan, and the number of published track sites is on the rise. We report a theropod assemblage from the Lower Jurassic Fengjiahe Formation in Xiyang, central Yunnan. It is the third report and description of dinosaur footprints from the Fengjiahe Formation, and this new track site is the largest in number of footprints for theropods in Yunnan. Over one hundred footprints are preserved on different layers of a claystone-dominated succession close to the Lower-Middle Jurassic boundary. The track area is referred to as a lacustrine shallow-water paleoenvironment. Tracks vary in size, morphology, and preservation. All are tridactyl and digitigrade, and some are identified as undertracks. The best preserved footprints were divided into three morphotypes: morphotype A (>8 cm–<21 cm) resembling *Grallator*; morphotype B (>27 cm–<30 cm) identified as *Kayentapus xiaohebaensis*; and morphotype C, an isolated footprint (39 cm) referred to the ichnogenus *Kayentapus*. Although footprint shape is influenced by many factors, biotic or abiotic, morphological differences among tracks such as size, divarication angles and proportions imply that at least three different kinds of theropods were visiting this site frequently. Theropod body fossils found in the surrounding area, such as *Sinosaurus*, turn out to be similar in body size to the projected size estimated from footprint lengths at the track site. In Yunnan, discoveries of theropod body fossils are rare. In that respect, the track record is a useful diversity indicator which can help to encompass theropod diversity patterns. Broadly speaking, large predators (five meters long or more) were uncommon in Early Jurassic ecosystems. Accordingly, large tracks are scarce on the track site, but not absent. Trackmakers of all sizes presumably coexisted in this tropical Jurassic ecosystem, and were regularly drawn to the track site in search of water or food resources.

## Introduction

Dinosaur footprints are valuable tools to infer dinosaur paleobiology and paleoenvironment. Track and trackway morphometrics can indeed provide important knowledge about biotic parameters such as speed and gait (Alexander, 1976; Gillette & Lockley, 1989; Thulborn, 1990), movement ability of the trackmaker (Coombs, 1980; Gatesy et al., 1999; Ezquerra et al., 2007), or sociality (Lockley & Matsukawa, 1999; Barco, Canudo & Ruiz-Omeñaca, 2006); and about abiotic parameters related to habitat (Gillette & Lockley, 1989; Lockley, 1991), biostratigraphy and paleobiogeography (Lockley et al., 2002; Matsukawa et al., 2005; Lucas et al., 2006; MacNaughton, 2007; Klein & Lucas, 2010). Dinosaur tracks have been found all over the world (Kuhn, 1963; Gillette & Lockley, 1989; Leonardi, 1994; Weishampel et al., 2004; Lockley & Matsukawa, 2009), including in China, which is rich in Triassic to Cretaceous paleoichnological material (Dong, 1992; Zhen et al., 1996; Li et al., 2010; Lockley et al., 2013). The first discovery of dinosaur footprints in China was made by the French scientist Teilhard de Chardin and the Chinese paleontologist Young in Shanxi Province, in 1929 (Teilhard de Chardin & Young, 1929). The resulting ichnotaxon was later coined *Sinoichnites youngi* by Kuhn (1958). The next discovery was made 12 years later in Liaoning Province (Yabe, Inai & Shikama, 1941). Subsequently, domestic and foreign scholars have conducted surveys and research on Chinese ichnofossils, and described a large number of vertebrate footprints (Young, 1960; Zhen et al., 1989; Dong, 1992; Matsukawa, Lockley & Li, 2006; Lockley et al., 2013; Li, 2015; Xing & Lockley, 2016). Nowadays, more than 40 dinosaur ichnogenera from about 60 localities have been reported in China, most of which are located in the Sichuan and Shandong Provinces (Chen et al., 2006; Lockley & Matsukawa, 2009; Lockley et al., 2013; Li, 2015; Xing, Lockley & Zhang, 2017; Xing et al., 2020; Xing et al., 2021).

Yunnan Province, in southwestern China, was originally renowned for its diverse Lufeng vertebrate faunas (Young, 1940; Young, 1951). Most dinosaur body fossils discovered in Yunnan are referable to sauropodomorphs (Young, 1941; Young, 1942; Young, 1947; Simmons, 1965; Yang, 1982; Chao, 1985; Bai, Yang & Wang, 1990; Dong, 1992; Zhang & Yang, 1995; Fang et al., 2000; Fang et al., 2004; Lü et al., 2006; Lü et al., 2007a; Lü et al., 2008; Upchurch et al., 2007; Sekiya, 2010; Xing et al., 2015; Wang et al., 2017; Zhang et al., 2018). At the same time the ornithischian and theropod records are relatively patchy (Young, 1948; Simmons, 1965; Xu, Zhao & Clark, 2001; Irmis, 2004; You et al., 2014; Wang et al., 2017). Yet, due to different preservation conditions, footprints and skeletons are often not preserved together or in the same proportions (Thulborn, 1990). Thus, the gap left by body fossils is often filled by trace fossils, which may provide an indicator of the diversity and distribution of a particular taxon in a given area. This is precisely the case for theropods in Yunnan Province: hitherto, merely seven theropod body fossils have been discovered, including relatively complete specimens such as *Panguraptor lufengensis* (You et al., 2014) and *Sinosaurus triassicus* (Young, 1948)*.* Meanwhile, multiple theropod ichnotaxa were described, including: *Changpeipus* (Young, 1960), *Eubrontes* (Hitchcock, 1845), *Grallator* (Hitchcock, 1858) and *Kayentapus* (Welles, 1971). That being said, the link between track morphotype and identity of the trackmaker is rarely straightforward because one genus can produce a variety of track morphotypes due to ontogeny or abiotic factors (Olsen, Smith & McDonald, 1998; Gatesy et al., 1999; Falkingham, 2014). At time of writing, over 10 track sites have been discovered in Yunnan Province. Two in particular, the Hemenkou and Yuanjitun track sites, both preserving sauropod tracks together with tracks from theropods, are large in surface area (120 m^2^ and 180 m^2^, respectively) and number of footprints (59 and 142, respectively; see Xing et al., 2016a; Chen & Huang, 1993; Xing et al., 2018).

In July 2018, a field team consisting of members of Yunnan University (Kunming), Institute of Vertebrate Paleontology and Paleoanthropology (Beijing) and George Washington University (Washington) investigated an Early Jurassic dinosaur track site originally discovered by local archeologist S. Hu in the 1990s. The locality, Xiyang, is located in Jinning County, Yunnan Province, and has never been officially reported. One hundred and twenty footprints were counted on several layers. This study intends to inspect the morphology of the tracks, categorize them, and discuss potential trackmakers and their paleoenvironment.

### Geological setting

**Figure 1:**
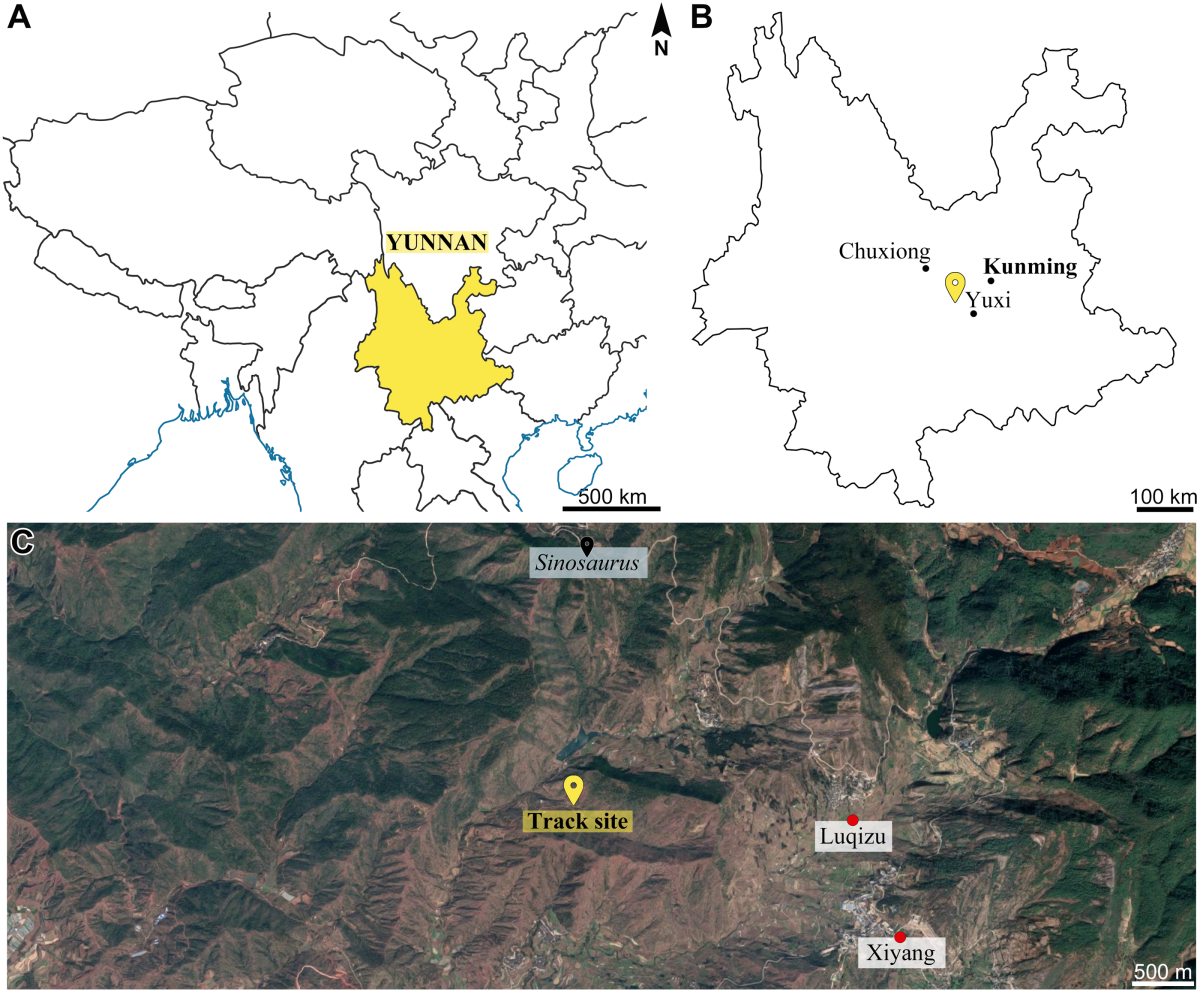
Geographical location of the Xiyang track site. (A) Partial map of southwestern China and surrounding areas, Yunnan Province is in yellow. (B) Map of Yunnan Province. (C) Satellite view showing locations of the Xiyang track site, nearby villages, as well as the site which yielded *Sinosaurus* KMV8701 (Image ©2021 CNES/Airbus, Landsat/Copernicus, Maxar Technologies).

**Figure 2:**
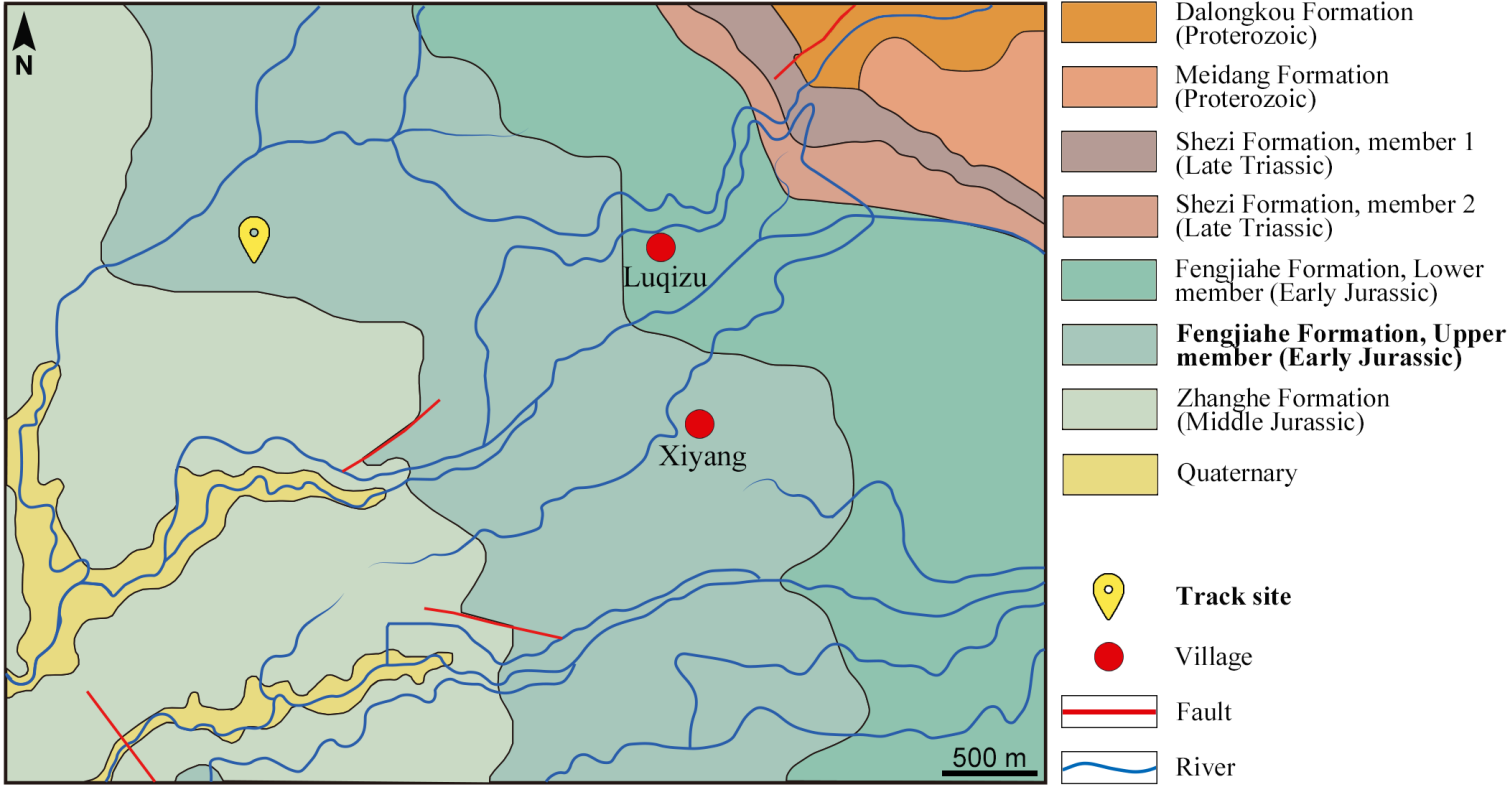
Geological map exhibiting the Xiyang track site as part of the upper member of the Fengjiahe Formation and its surroundings. Based on the geological map of Yunnan Institute of Geological Survey (1989). (Since then, Fang et al. (2008) revised the stratigraphical nomenclature of the whole area, leading to the identification of sections of the Shezi Formation as Yubacun Formation.)

The footprint assemblage was found by the mountain path near Xiyang Village, Xiyang Township, Jinning County, Kunming Prefecture, Yunnan Province, China (Fig. 1). The Xiyang area is situated in the Chuxiong subregion. It is dominated by the Lower Jurassic Fengjiahe Formation but outcrops of the Middle Jurassic Zhanghe Formation are exposed towards the eastern part, in the vicinity of the track site (Bureau of Geology and Mineral Resources of Yunnan Province, 1990; Fang et al., 2000; Cheng et al., 2004; Fang et al., 2008; Yunnan Institute of Geological Survey, 2021, pers. comm.; Fig. 2). As a matter of fact, stratigraphic investigations on the field have shown that the Lower-Middle Jurassic boundary is located 33 m above the track site, thus confirming that the track-bearing layers are part of the upper member of the Fengjiahe Formation (Fig. S1). The Fengjiahe Formation is conformably underlain by rocks of the Lower Jurassic Yubacun Formation (formerly Shezi Formation, redefined by Fang et al., 2008) and conformably overlain by the Middle Jurassic Zhanghe Formation (Fang et al., 2008). The Fengjiahe Formation has a variable total thickness, especially in the east–west direction; the western part is generally over 1,500 m thick, yet the eastern part is less than 1,000 m (Wang et al., 2019). Although Xu, Zhao & Clark (2001) and Hu (1993) referred to Early Jurassic deposits in Jinning County as the Lower Lufeng Formation, Fang et al. (2000) and Fang et al. (2008) revised the stratigraphical nomenclature in order to distinguish the sedimentary characteristics of Chuxiong and Kunming subregions. As a result, the term Lufeng Formation should no longer be used in the Chuxiong subregion, which is on the west side of Kunming subregion. Hence, in the former, the sequence of Early to Middle Jurassic lithostratigraphic units is as follows: Yubacun Formation, Fengjiahe Formation, Zhanghe Formation and Shedian Formation (Fig. 2) and in the latter, the same units are generally regarded as Yubacun Formation, Lufeng Formation, Chuanjie Formation and Laoluocun Formation (Fang et al., 2008; Pang, 2010). Based on sedimentological evidence, the Chuxiong and Kunming subregions represent contemporaneous deposits differing in terms of environments and characteristics (Cheng et al., 2004; Fang et al., 2008; Pang, 2010). Chuxiong subregion is represented by a larger lacustrine sedimentary paleobasin with deeper water and much more stable lower-energy fluvial processes, while Kunming subregion is characterized by small basins scattered around great lakes (Cheng et al., 2004; Fang et al., 2008). According to Deng et al. (2017: fig. 3, fig. 4), the track site area was tropical to subtropical during the Early Jurassic. The reddish claystone and mud cracks observed on the track-bearing surfaces support this assessment. Such a climate could cause the lakes to undergo periodic droughts or floods, leading to suitable environmental conditions to preserve tracks (Paik, Kim & Lee, 2001).

**Figure 3:**
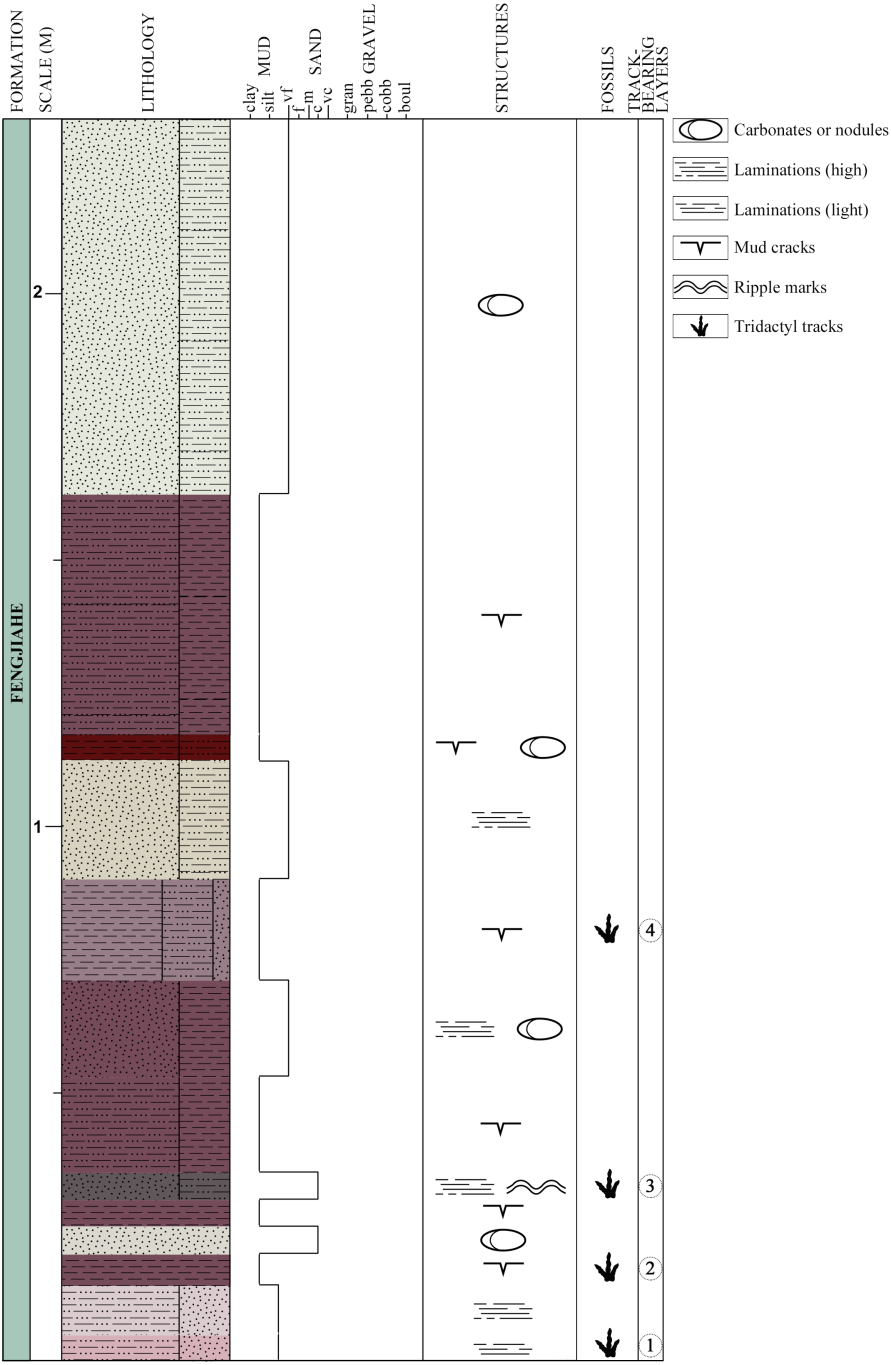
Schematic lithological and stratigraphic section of the Xiyang track site. Colors of the lithological units are based on the colors observed on the field.

**Figure 4:**
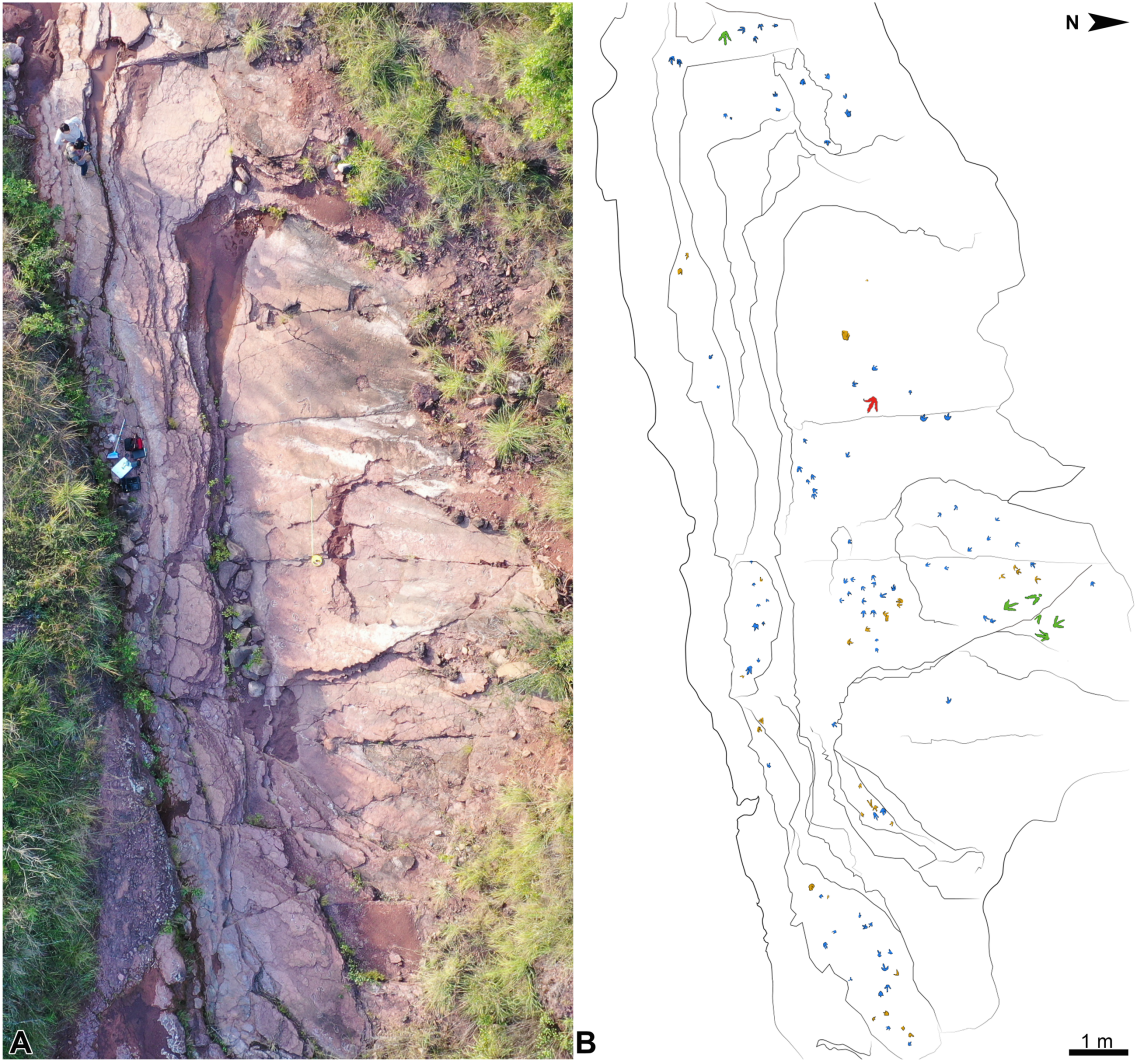
Overview of the Xiyang track site. (A) Photograph of the whole inclined track-bearing outcrop. (B) Outline drawing showing the distribution of the tracks. Morphotypes A, B and C are painted in blue, green, and red, respectively; unidentified tracks appear in brown.

Thirteen beds are exposed at the locality, four of which display tridactyl tracks (three claystone layers and one sandstone-dominated layer, Fig. 3). They are labeled chronologically as layers 1–4. Layer 1, the oldest, is composed of light reddish and grey siltstone mixed with very fine sandstone. It is lightly laminated, with mud cracks on its surface. Layer 1 bears the tracks XIY-105, 106-L1 and 106-R2. Layer 2 bears tracks XIY-052 and 054 to 107. It consists of dark reddish-brown silty claystone with mud cracks that is in conformity with layers 1 and 3. Layer 3 is made of dark grey to brown coarse sandstone, mixed with siltstone. It is finely laminated, and exhibits ripple marks on the exposed upper bedding plane surfaces. Layer 3 bears tracks XIY-045 to 051 and XIY-053. Layer 4 contains tracks XIY-001 to 044 and XIY-108 to 112. It is composed of reddish silty claystone interbedded with greyish fine sandstone layers, and exhibits mud cracks. The thirteen exposed beds of the Fengjiahe Formation have a total thickness of 227 cm and a strike of N. 35° W. The sequence mainly consists of reddish muddy siltstone, alternating with dark red silty claystone and a few beds dominated by grey yellowish sandstone. Some beds show horizontal laminations, and carbonates or nodules (of approximately 5 cm of diameter) are visible in a few layers (Fig. 3). Sedimentary structures, such as mud cracks and low amplitude ripple marks (average spacing of 1.5 cm, see Fig. S2), prove that water and hence, soft and wet paleosubstrate, were present. The composition of sedimentary rocks, in turn, implies that the setting was relatively stable, low in energy and in shallow water. Consequently, the Xiyang track site was most likely formed in and adjacent to a lacustrine paleoenvironment (Bureau of Geology and Mineral Resources of Yunnan Province, 1990; Fang et al., 2008). Further sedimentological and geochemical analyses of the area or basin were not considered for reasons of time and technical means and are, in all cases, beyond the scope of this paper.

The paleofauna in Chuxiong subregion is not as rich as in Kunming subregion, which was a more suitable living area (Fang et al., 2008). Nevertheless, it should be stressed that suitable living area does not necessarily equal preservation potential. The Early Jurassic fauna in Yunnan is represented by the *Lufengosaurus* fauna. In the Fengjiahe Formation, both sauropodomorph and theropod dinosaurs have been discovered and include *Sinosaurus* near the Xiyang track site (Hu, 1993; Peyre de Fabrègues et al., 2020; Fig. 1). Several theropod track sites have also been reported in the vicinity (Zhen et al., 1986).

At the Xiyang track site, one hundred and twenty tridactyl true tracks and undertracks are preserved on a large inclined outcrop, which represents the largest number of theropod footprints discovered in Yunnan so far (Fig. 4; Fig. S3). This is the third discovery of dinosaur tracks in the Fengjiahe Formation (Zhen et al., 1986; Xing et al., 2016b). Several ichnotaxa, including *Anomoepus, Eubrontes, Grallator*, *Kayentapus* and the now controversial *Zhengichnus,* were previously reported from Fengjiahe Formation (Zhen et al., 1986; Chen et al., 2006; Lockley et al., 2013; Xing et al., 2016b).

## Materials & Methods

The track site remained unstudied for over two decades and underwent continuous weathering and erosion. Since 2018, some tracks have been severely eroded and a few even vanished because of breakage of some of the most friable rock layers. We will here follow Gatesy & Falkingham (2017) to describe the state of preservation of the tracks, and Marchetti et al. (2019) to assign grades (on a scale from 0 to 3) evaluating the morphological preservation. Given the layout of the track site (Fig. 4), it is most likely that there are still many undiscovered footprints. During the last mission in early 2021, removal of one broken superficial layer exposed a set of previously concealed footprints.

The whole track-bearing outcrop is approximately 20 m in length and 7 m in width (Fig. 4). A schematic lithological and stratigraphic section was drawn using SedLog (version 3.1) and edited using Adobe Photoshop CC 2019. One hundred and twenty tracks, including 1 trackway and 5 track associations comprising 14 tracks, were identified on the exposed layers and cataloged under collection numbers XIY-001 to XIY-112 (tracks from the same trackway or track association share the same number). To facilitate field observation and study, they were outlined with chalk and serially numbered following an East-West axis and from the top to bottom layers (XIY-001 is on the top layer, labeled as layer 4; see Fig. S3). The entire track site was traced on transparent plastic film, and tracks were not collected. Authorizations to work on the field were given verbally by Mr. Deke Zhao for the Culture and Tourism Bureau of Xiyang Township.

With only a few track associations and one trackway preserved, it is difficult to identify whether isolated tracks were made by a right or left pes. To address this matter, we follow Thulborn’s (1990) comment about digital pads: the metatarsophalangeal pad posterior to digit IV is always higher than that of digit II. The number of phalangeal pad impressions can also be used as a discriminating parameter when footprints do not preserve metatarsophalangeal pads. Generally, the typical phalangeal pad formula for theropods is x-2-3-4-x (Thulborn, 1990: fig. 5.4), even if a distinction should be made between the foot skeleton and its configuration and the impression of the foot and its tissues (Gatesy & Falkingham, 2017).

True tracks were distinguished from undertracks based on Milàn & Bromley (2003), Milàn & Bromley (2007) and Milàn (2006). Undertracks always appear broader and less well defined than true tracks. Generally, they tend to become shallower and more vaguely defined at successively lower levels (Thulborn, 1990). However, without a vertical view and in the absence overlying layers, undertracks are only tentatively identified here.

Photos of individual footprints and the track site were taken with a Canon EOS 5D Mark II camera and a DJ Mavic 2 Pro drone. Photogrammetric photos of one track (XIY-048) were captured using a Nikon D5200 camera. This particular track was chosen because it is the largest of the track site, it is well preserved and well situated on the outcrop (not against an edge). The 3D model was generated and then modified using the software Zephyr Pro (version 4.530). Data were archived on MorphoSource.org (doi.org/10.17602/M2/M360516). Using the 3D model, a false-color depth map was generated in Paraview (version 5.9.0), with the elevation function. Interpretative outline drawings of the track-bearing surface and of isolated tracks were done at the Yunnan University using Adobe Illustrator CC 2019 and Adobe Photoshop CC 2019. On-site measurements were taken using a measuring tape, and additional measurements were taken from digital photographs using ImageJ (version 1.8.0; Schneider, Rasband & Eliceiri, 2012).

For each track, the subsequent measurements were made following Thulborn (1990: figs. 4.8, 4.9, 4.11): length (L), width (W), length of digits (LD, measured from the tip of the digit to the rear margin of the posterior most phalangeal pad or from the tip to the point midway between the hypex, depending on the presence of the metapodium) and divarication angles (taken between the midline of each digit; see Fig. S4). For the trackway: pace length (PL), stride length (SL), and pace angulation (PA) were measured (Fig. S4). Finally, track length to width (L/W) and projection ratios were calculated. Both ratios, especially the projection ratio (*i.e.*, digit III projection beyond digit tips II and IV; TE in Fig. S4) can be used to determine morphological variation in theropod tracks (Olsen, Smith & McDonald, 1998; see Fig. S4). Projection ratios can be calculated in a variety of ways. One of the most prevalent methods is used by Li (2015: p. 9; herein PR). Still, different authors tend to use different equations, thus making comparisons difficult. In order to propose more accurate values and to make valuable comparisons we also used the “corrected” projection ratio (compensating for the divarication angles) following Olsen, Smith & McDonald (1998: p. 586; herein CPR). Based on the aforementioned measurements, bivariate plots of track length versus track width and track length versus projection ratio, as previously applied by Demathieu et al. (2002), Romano & Whyte (2003) or Sciscio et al. (2017), were generated in Microsoft Excel 2019.

Hip height (H) of the trackmaker was calculated using morphometric ratios based on track length (L). We used two approaches to observe potential variation in the results:

Following Thulborn (1990), for small theropods (L < 25 cm) *H* = 4.5 × *L*, for large theropods (L > 25 cm) *H* = 4.9 × *L*

Following Alexander (1976), for all theropods *H* = 4 × *L*

Body length (BL) of the trackmaker was calculated following Paul (1988), as applied by Weems (2006a: figs. 4B, 9B) and Sciscio et al. (2017):

When L < 35 cm *BL* = 4 × *H*, and when L ≥ 35 cm *BL* = 2 × *H* + 3.5

Gait of the trackmaker was estimated by measuring the stride length (SL) to hip height (H) ratio (SL/H). According to Alexander (1976), followed by Thulborn & Wade (1984), dinosaurian gaits are classified into three categories: “walk” (SL/H ≤ 2.0), “trot” (2.0 < SL/H < 2.9) and “run” (SL/H ≥ 2.9).

### Descriptive Ichnology

#### Isolated tracks

**Figure 5:**
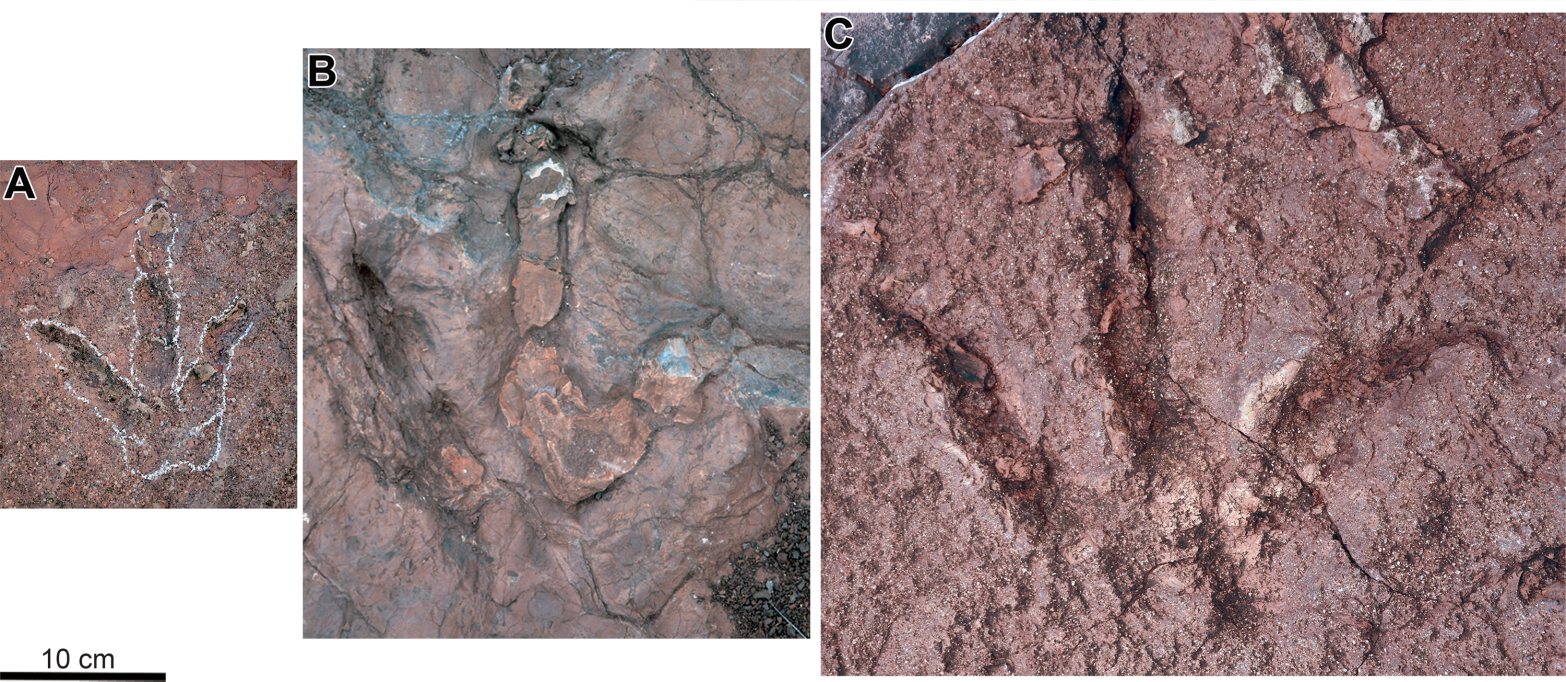
Photographies of well-preserved tracks for each morphotype identified at the Xiyang track site. (A) XIY-053 from morphotype A. (B) XIY-108 from morphotype B. (C) XIY-048 from morphotype C.

All the tracks are tridactyl and digitigrade, and show no preferred orientation. Most of them are true tracks and some, mostly large ones, are regarded as undertracks due to their less well-impressed digits and lack of edge definition (XIY-005, 033, 070, 071, 074, 075, 076, 107). Large animals usually tend to leave more undertracks due to their heavier weight (Thulborn, 1990; Lockley, 1991). Metatarsophalangeal pads are imperfectly preserved or indistinct in most tracks, and phalangeal pads are sometimes difficult to observe. Footprints range from 8 cm to 39 cm in size (Table S1, Fig. 4, Figs. S5–S6) and from 0 to 2.5 in degree of morphological preservation (Marchetti et al., 2019; Table S2). Tracks on different layers did not show consistency in preservation. Even on the same layer, the preservation (especially depth) of different tracks varies variation due, in part, to substrate consistency. Based on their size and shape, we subdivide the tracks into three morphotypes: A, B and C (Fig. 5). Thirty-five tracks are not attributed to any morphotype (Table S1) because of their poor state of preservation (grades ranging from 0 to 1, see Table S2).

#### Morphotype A

Morphotype A contains 77 tracks, which represent 64% of described tracks, including several track associations and one trackway (Table S1). A few tracks appear to be undertracks (track numbers noted above). Footprints are small to medium-sized (8 to 21 cm, mean 14.5 cm), with a medium divarication angle IIˆIV (24° to 111°, mean 50°), an average L/W ratio of 1.2, an average PR of 1.6, an average CPR of 1.9 and relatively strong mesaxony and symmetry (Table 1). They all have slender and straight digit impressions. Claw impressions are mostly observed in this morphotype, while metatarsophalangeal pads are apparent on some tracks and phalangeal pads are, in a general way, not discernible (Figs. S7A–S7E). According to some relatively well-preserved tracks, such as XIY-053 and XIY-065, we infer that the phalangeal pad formula is x-2-3-4-x.

**Table 1:** Mean measurements for each morphotype from the Xiyang track site, Yunnan Province, China.

Morphotypes	L	W	II-IV	L/W	PR	CPR
A	14.5	11.8	50	1.2	1.6	1.9
B	28.1	25.6	68	1.1	2.1	4.3
C	38.8	40.4	74	0.96	1.6	3.1

**Notes.**

Abbreviations following the order of the table: L maximum length (cm) W maximum width (cm) II-IV angle between digits II and IV (degrees) L/W length over width ratio PR projection ratio following Li, 2015 (p. 9) CPR “corrected” projection ratio following Olsen, Smith & McDonald (1998) (p. 586)

Among all morphotype A tracks, XIY-053 is the best preserved with impressions of phalangeal and metatarsophalangeal pads (Fig. 5A). It is an isolated left pes with a length of 15.5 cm and a width of 12.7 cm (L/W = 1.22). Digits are slender and taper distally. Claw impressions are blunt, but phalangeal pads are well delimited and conform to the formula given above. Phalangeal pads are oval, and those of digit IV are distinctly smaller than those of digit III. The divarication angle IIˆIII (20°) is smaller than that of IIIˆIV (40°). The metatarsophalangeal area is clearly visible at the posterior part of the track. Three metatarsophalangeal pads of similar size and shape are preserved at the proximal ends of digits II, III, and IV. The posterior margin of the metatarsophalangeal pad behind digit IV extends slightly more posteriorly than that behind digit II.

#### Morphotype B

Morphotype B includes 7 tracks (Table 1). Most are identified as undertracks with only two exceptions (XIY-087 and 108) (Fig. 5B). Four of them are incomplete or indistinct in outline (XIY-070, 74, 75, 76). They are larger (27 to 30 cm, mean 28.1 cm) and have a higher average divarication angle IIˆIV (62° to 70°, mean 68°) than morphotype A, an average L/W ratio of 1.1, an average PR of 2.1 and an average CPR of 4.3 (Table 1). Tracks assigned to morphotype B are mesaxonic and subsymmetrical. The digits are elongated, with well-delimited phalangeal pads in some tracks (Figs. S7F–S7G). Metatarsophalangeal pad impressions are generally not obvious.

The track XIY-071 is the most complete for morphotype B (Fig. S7G). It is a medium-sized track, which is 30 cm long and 26 cm wide (L/W = 1.15). Digit III, anteriorly directed, is the longest digit and digit II is the shortest. Two sharp claw impressions can be observed on digits II and IV. An oval claw impression is observed at the tip of digit III, most likely the result of a slight forward shift in body weight (Wilson, Marsicano & Smith, 2009). Except on digit III, phalangeal pads are not well defined. Still, we observe the presence of at least three phalangeal pads on digits III and IV and hence, a possible phalangeal pad formula x-2-3-4-x. The divarication angle IIˆIV is high (70°), and IIˆIII (39°) is only slightly higher than IIIˆIV (31°). A relatively large and V-shaped metatarsophalangeal pad impression is visible on the posterior part of the footprint.

#### Morphotype C

Only one track is referred to Morphotype C (Table 1; Table S1; see the 3D model in supplementary material): an isolated right pes identified as the largest track of the assemblage (Fig. 5C; Fig. S8). XIY-048 is 39 cm in length and 40 cm in width (L/W = 0.96), with a PR of 1.6 and a CPR of 3.1. All three digits are relatively slender, and no hallux trace is observed. Digit IV shows a strong outward curvature relative to the track midline, while digits III and II are relatively straight. Digit II imprint is deeper. Digit III is the longest digit, and digit IV appears to be the shallowest. Sharp claw impressions are visible on all three digits. The divarication angle IIˆIV is high (74°). The divarication angle IIIˆIV (38°) is slightly superior to IIˆIII (34°). Phalangeal pads are hardly noticeable, although it seems that there are three phalangeal pads on digit III and two on digit II. Two metatarsophalangeal pads are preserved posteriorly to digits II and IV. The metatarsophalangeal pad behind digit IV is more extended posteriorly than that of digit II.

In summary, morphotype A is small to medium in size with the lowest average divarication angle IIˆIV, morphotype B is medium with an intermediate average divarication angle IIˆIV, and morphotype C is large with a high divarication angle IIˆIV. The overall morphology of each morphotype is quite distinct, with CPR of, respectively, 1.9, 4.3 and 3.1.

### Track associations and trackway

Five track associations and one trackway are formed of 14 tracks, all assigned to morphotype A (Table S1). They are identified as such based on their alignment, preservation state and the consistency of the shape and size of the footprints. Track associations (TA1-5) comprise two tracks only and the trackway (TW1) comprises four tracks (Figs. S9–S13). Most tracks are considered as true tracks. The tracks are distributed on different stratigraphic levels without preferred orientation. The track associations and trackway show a narrow width and gauge.

TW1 consists of four small and distorted tracks, the first one of which is particularly incomplete (XIY-028-L1 & R1, XIY-028-L2 & R2; Fig. S9). The track morphology is similar to that of TA1. The most complete footprint is track XIY-028-R2, the fourth impression of the trackway, which has a length of 11.7 cm. Divarication angles IIˆIV are consistent, between 43° and 50°. The trackway displays a total length of 129 cm, with an extreme narrow gauge like TA1. TW1 has an average PL of 42 cm, an average SL of 84.5 cm, and PA is 175.5° on average. Using the equation given by Thulborn (1990: p.86), the obtained PA is 171°. All four footprints are relatively straight with respect to the trackway midline. TA1 and TW1 are on the same layer and show a similar morphotype and direction, but TA1 tracks are distinctly larger than those of TW1 (Fig. S9).

TA1 includes two medium tracks (XIY-026-R1 & L1; Fig. S9) with phalangeal pads, but no complete metatarsophalangeal pad visible. The footprints are 17.6 and 20.7 cm long. Surprisingly, the divarication angle IIˆIV is much dissimilar from the right pes to the left pes (52° and 24°, respectively). We put this down to the poor preservation state of the right pes. The association has a total length of 113 cm and an extremely narrow gauge; the left and right pes are practically on the same line.

TA2 consists of two weakly impressed medium-sized footprints (XIY-042-R1 & L1; Fig. S10), which are most likely undertracks. The footprints are 15.6 cm and 16.5 cm long. The divarication of outer digits is 38° and 29°. The total length of the succession is 50 cm, and the gauge equals 6.5 cm. The pace length is 46 cm.

TA3 comprises two poorly preserved medium-sized footprints (XIY-047-R1 & L1; Fig. S11). The tracks are both 19.2 cm in length. The divarication angles IIˆIV are rather high: 64° and 65°. TA3 has a total length of 80 cm and a very narrow gauge. The pace length is 58 cm, which is longer than TA2 and consistent with the size of the tracks.

TA4 consists of two rather small tracks with slender digits, but no metatarsophalangeal pad impression (XIY-060-L1 & R1; Fig. S12). They are most likely penetrative tracks based on the extremely thin digit impressions, lack of digit pads, claw impressions, and presence of mud bulges between the digits (Gatesy & Falkingham, 2020). The footprints are 11 cm and 11.5 cm in length and present high divarication angles IIˆIV of 66° and 111°. The total length of the association is 56 cm. The gauge is narrow and the pace length is 44 cm.

TA5 consists of two poorly preserved small to medium-sized tracks (XIY-106-L1 & R1; Fig. S13). Footprints are 13 cm and 14 cm in length. The divarication angles of outer digits are 51° and 74°. The association has a total length of 64 cm, with a very narrow gauge of 3.6 cm. The pace length is 42 cm.

## Discussion

### Ichnotaxonomy

Theropod tracks at the Xiyang track site offer a variety of sizes, morphologies and were impressed on substrates of differing rheologies offering different preservation conditions. Tracks from this locality are attributed to theropod dinosaur trackmakers based on the tridactyl morphology, absence of metatarsal impressions, proportions (length exceeding width, except for morphotype C), length of digit III (longer than digits II and IV), and claw impressions (Hitchcock, 1845; Thulborn & Wade, 1984; Lockley, 1991; Lockley, Matsukawa & Li, 2003).

Theropod tracks are globally very common ichnofossils in Jurassic-aged geological layers. The most abundant ichnocoenoses are known from North America (Lockley & Hunt, 1995), where ‘historical’ ichnotaxa, such as *Eubrontes* (Hitchcock, 1845) and *Grallator* (Hitchcock, 1858), were first described. For the period from the Carboniferous to the Early Jurassic, despite the continental break up during the Jurassic, vertebrate ichnofaunas appear to remain relatively cosmopolitan globally (Haubold, 1984; Lockley & Hunt, 1996). This is reflected in the fact that most Chinese ichnotaxa are based on type material from other countries (mostly United States of America, see Lockley et al., 2013). Since the 1940s, thousands of theropod tracks have been reported in China, and hundreds formally named (Zhen et al., 1989; Li, 2015). Their taxonomic diversity has, however, clearly been over-interpreted given that many new ichnogenera and ichnospecies were erected based on poorly diagnostic material or elusive diagnoses (Lockley et al., 2013). As a result, comparisons and identifications of newly discovered tracks and trackways have been seriously hampered. In recent times, naming theropod tracks, especially those abundantly represented in Jurassic deposits and associated with the *Anchisauripus*-*Eubrontes*-*Grallator* plexus (Olsen, Smith & McDonald, 1998), has proven to be particularly challenging (Xing et al., 2016a). Lockley et al. (2013), followed by Li (2015), attempted to address this ‘splitting’ issue by pruning a number of ichnogenera. Lockley et al. (2013) mostly removed Jurassic ichnotaxa, shrinking the number of valid ichnogenera from 23 to 9. We here mainly follow their work, apart from *Zhengichnus jinningensis* (BPV-FP7; Zhen et al., 1986) from the Fengjiahe Formation of Yunnan (considered as a *nomen dubium* by Lockley et al., 2013) that we included to our comparative data because of its provenance. Likewise, *Eubrontes pareschequier* and *Eubrontes carbonicus* are here regarded as synonyms of *Changpeipus carbonicus* following Xing et al. (2014a). These considerations lead to a total of 10 valid ichnogenera of theropod tridactyl tracks described from the Jurassic of China (Table S3).

Interestingly, while the most widespread ichnotaxa assigned to theropod trackmakers, such as *Eubrontes*, *Grallator* or *Kayentapus*, can be found in any Jurassic strata in China, they are mostly restricted to Upper Triassic to Lower Jurassic layers in other continents, particularly North America where they constitute key elements of ichnofaunas (Lockley & Hunt, 1995; Lucas et al., 2006).

In Yunnan Province, a number of footprints are known from Lower to Upper Jurassic layers. Non-avian theropod tracks of all sizes are unambiguously predominant (Lockley et al., 2013), but some avian theropod, ornithopod, sauropod and thyreophoran tracks have been reported as well (Xing et al., 2014b; Xing et al., 2016b; Xing, Lockley & Romilio, 2019; Xing et al., 2019). The theropod track record in the Jurassic of Yunnan includes 5 ichnogenera: *Changpeipus* (Young, 1960), *Eubrontes* (Hitchcock, 1845), *Grallator* (Hitchcock, 1858), *Kayentapus* (Welles, 1971), and *Zhengichnus* (Zhen et al., 1986). Apart from *Changpeipus,* which is known from the Lufeng Formation (Xing et al., 2009), the four other ichnogenera were reported from its lateral equivalent, the Fengjiahe Formation. The Fengjiahe Formation of Jinning County, where Xiyang track site is located, yielded 6 ichnospecies: *Eubrontes monax* (formerly *Paracoelurosaurichnus monax*), *Eubrontes platypus*, *Eubrontes xiyangensis* (formerly *Youngichnus xiyangensis*), *Grallator limnosus*, *Kayentapus xiaohebaensis* (formerly *Schizograllator xiaohebaensis*), and *Zhengichnus jinningensis* (Zhen et al., 1986).

At Xiyang track site, tracks of morphotype A are small to medium in size. Based on the above-mentioned description, they cannot be referred to one of the ichnospecies already reported from the Fengjiahe Formation (Table S4). They nonetheless seem to be part of the *Anchisauripus*-*Eubrontes*-*Grallator* continuum (Olsen, Smith & McDonald, 1998). We herein regard *Anchisauripus* as a synonym of *Grallator*, considering that the only discriminating character given by Olsen, Smith & McDonald (1998) is the size of tracks (PR, L/W ratios, and divarication angles of both type specimens being extremely similar) and following Baird (1957), Weems (1992) and Weems (2019).

**Figure 6:**
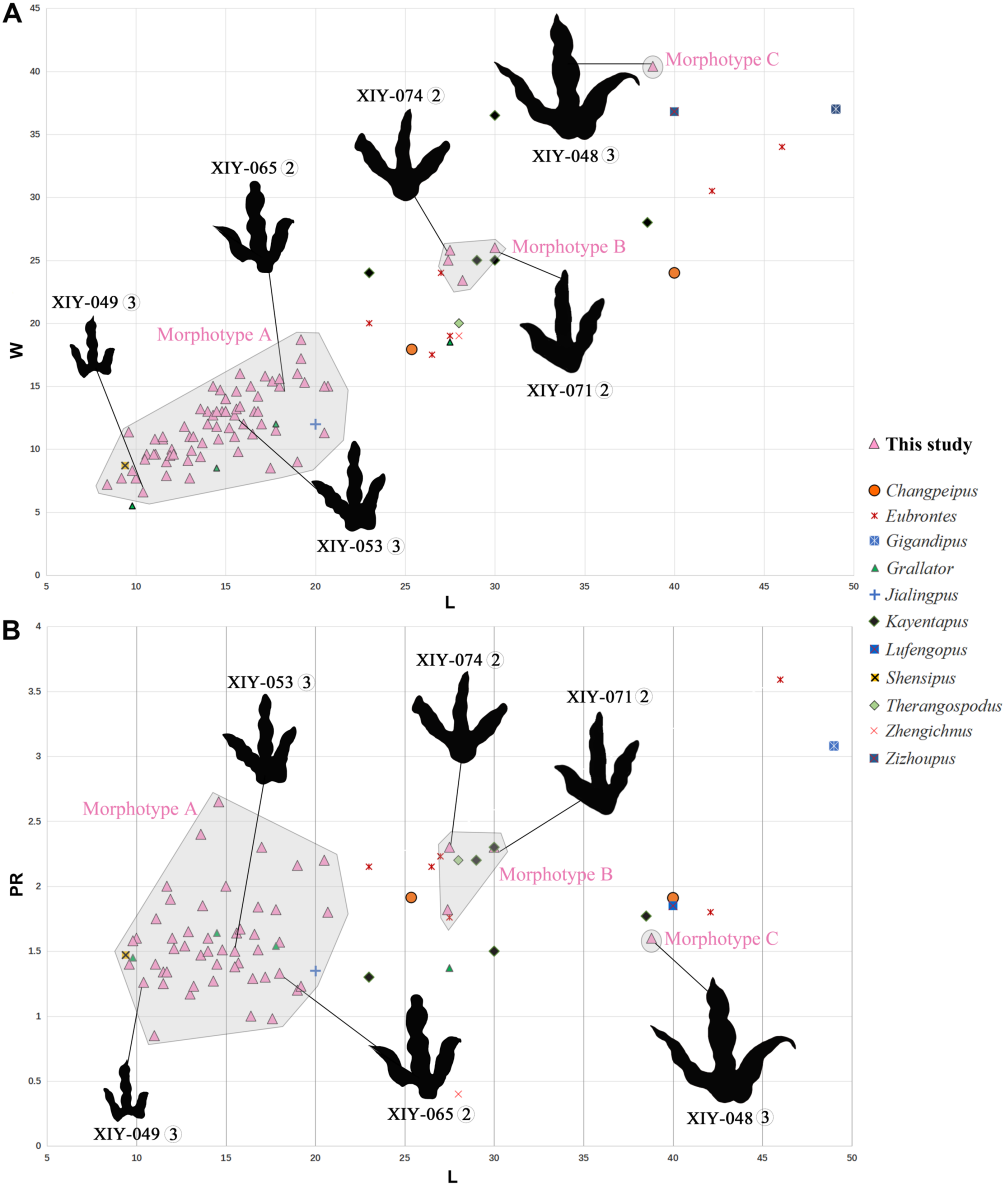
Bivariate plots illustrating morphological variability. Plots (A) of length function of width and (B) length function of projection ratio showing the morphological variability of the three morphotypes from the Xiyang track site relative to holotypes of theropod tracks from the Jurassic of China (except *Therangospodus* and *Gigandipus* which are the paratypes, see Table S1 for ichnospecies and specimen numbers). Measurements of all tracks follow Thulborn (1990) and projection ratios (PR) are calculated as described by Li (2015). Circled numbers next to track number refer to the track-bearing layer.

**Figure 7:**
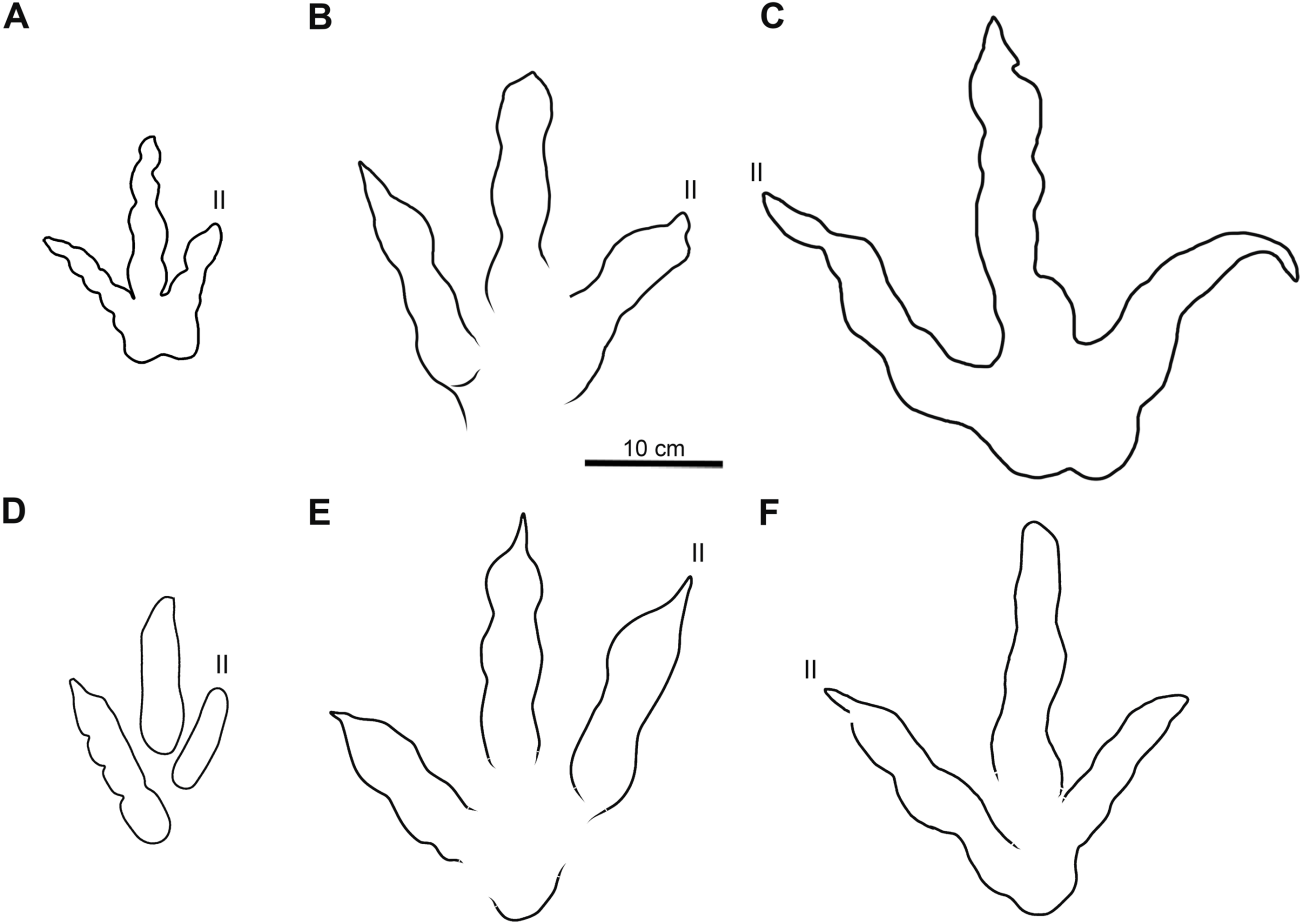
Comparative line drawings of Xiyang tracks and most similar theropod tracks from the Jurassic of China. (A) XIY-053 (morphotype A). (B) XIY-108 (morphotype B). (C) XIY-048 (morphotype C). (D) *Grallator yemiaoxiensis* (CFNY8, mirrored), based on Yang & Yang (1987). (E) *Kayentapus xiaohebaensis* (BPV-FP4), based on Zhen et al. (1986). (F) *Kayentapus hopii* (GLS-T1-R2), modified from Xing et al. (2020). All tracks at the same scale.

Tracks of morphotype A show affinities with the ichnogenus *Grallator*. However, it is to be noted that *Grallator* tracks tend to be relatively long: the original diagnosis of the ichnogenus (Lull, 1904) states that the L/W ratio should be equal or higher than 2, a condition not observed in the morphotype A from Xiyang (average L/W of 1.2). This could be explained by the absence of a proper metatarsophalangeal pad impression in most morphotype A tracks or other factors, such as the speed of the animal, the substrate, the substrate rheology, etc. *Grallator* footprints are generally narrow: following the diagnosis, the divarication angle of outer digits (IIˆIV) is between 10° and 30°. On average, the morphotype A from Xiyang presents a higher divarication angle of outer digits (50°) than the type specimen of *Grallator* (Hitchcock, 1858; Olsen, Smith & McDonald, 1998). In this regard, it is closer to the ichnogenus *Eubrontes*, for which the original diagnosis gives a divarication of outer digits around 30° to 40° (Hitchcock, 1845). Some ichnospecies referred to *Eubrontes* even display a divarication approximating 50° (e.g., *E. glenrosensis*, *E. nianpanshanensis*, *E. zigongensis;* in Li et al., 2010; Yang & Yang, 1987; Gao, 2007). Notwithstanding, use of the divarication angle of outer digits as a discriminating character is questioned by some authors, such as Li (2015), considering that some tracks with a high divarication angle were labelled as *Grallator* in the past, and that it can be a function and influenced by other factors, like rheology. That being said, and despite the fact that the calculated mean (50°) and median (49°) demonstrate that most morphotype A tracks have a divarication angle IIˆIV close to 50°, it is important to stress that there is considerable variation in the latter (angles ranging from 24° to 111°; see Table S1). This variation might be function of the depth of the track, dependent on both the speed of the trackmaker and sediment consistency (Milàn, 2006). Based on the small to medium size of morphotype A tracks and both calculated projection ratios, we infer that they are grallatorid tracks. The closest footprints in terms of morphology appear to be two ichnospecies referred to *Grallator*: *G. wuhuangensis* from Sichuan Province and *G. yemiaoxiensis* from Chongqing city, which have rather unspecific diagnoses and are poorly illustrated in the literature (Yang & Yang, 1987). This hypothesis is reinforced by the data distribution observable in bivariate plots, in which we implemented length function of width (Fig. 6A), and function of PR (Fig. 6B). On this basis, and in the absence of genuine diagnostic characters allowing a referral to either *G. wuhuangensis* or *G. yemiaoxiensis*, morphotype A is simply identified as *Grallator*-like (Fig. 7D). Observations made on TW1 support this conclusion. The morphotype A trackway shows a narrow gauge and relatively large pace and stride lengths, which coincides with all *Grallator* diagnoses (Hitchcock, 1858; Lull, 1904; Zhen et al., 1986; Yang & Yang, 1987; Table S1). Because *Grallator*-like and *Anomoepus*-like tracks are commonly found in Lower Jurassic deposits, some Chinese studies tended to confuse them for one another (Lockley & Matsukawa, 2009). *Anomoepus*, an ornithopod ichnogenus previously identified in the Fengjiahe Formation (Xing et al., 2016b), consists of small (<20 cm) tridactyl tracks with a wide divarication angle and a high CPR (>2; see Olsen & Rainforth, 2003). Except for the wide divarication angle of some tracks, morphotype A does not display *Anomoepus*-like characters. In *Anomoepus*, digit pads are often separated by two creases, digits are relatively robust and PR is high (Olsen & Rainforth, 2003; Xing et al., 2016b), while morphotype A has a single crease, rather gracile digits and a lower PR (although these two last characteristics may be related to the preservation of the substrate).

Tracks of morphotype B are average in size. Based on the description above, morphotype B seems to present affinities with *Changpeipus* or *Kayentapus*, of which one ichnospecies is represented in the Fengjiahe Formation.

The tracks of morphotype B display affinities with the type-specimen of *Changpeipus* (V 2472.2; Young, 1960): they show comparable size and divarication angle of outer digits. *Changpeipus* is represented in the Lower Jurassic of Yunnan by two tracks that were not proven to be part of the same trackway, even if their relative position suggests a single step (Xing et al., 2009). These two tracks resemble morphotype B, but with a relatively lower divarication angle of outer digits, a pad formula of x-2-3-2-x (Xing et al., 2009 *contra* Lockley et al., 2013) and a swollen distal pad of digit III. As a matter of fact, the original diagnosis of *Changpeipus* mentions the increase in size of the phalangeal pads towards the distal end of digit III, as well as a digit IV projecting further anteriorly than digit II and exceeding the latter in length (Young, 1960). These characteristics are not observed in morphotype B tracks. Moreover, the PR of *Changpeipus* (1.9) is close to morphotype B (2.1), but the CPR (3) does not match (4.3). *Eubrontes*, a common ichnogenus of large-size belonging to the same family than *Changpeipus*: Eubrontidae (Lull, 1904), is quite similar to the latter in general morphology. Some specimens assigned to the ichnogenus *Eubrontes* appear to fall within the scope of morphotype B in bivariate plots (Fig. 6), but several diagnostic features of *Eubrontes* are non-existent in morphotype B: L/W ratio around 1.5, projection of digits II and IV along the axis of digit III about equal, and divarication angle of outer digits between 30° and 40° (Hitchcock, 1845; Olsen, Smith & McDonald, 1998). Two other ichnogenera appear close to morphotype B in the plots: *Kayentapus* and *Therangospodus* (Fig. 6). Referral to *Therangospodus* is discarded based on digit diagnostic features, particularly the absence of coalesced, elongate, oval phalangeal pads, not separated into discrete phalangeal pads (Lockley, Meyer & Moratalla, 1998; Xing, Harris & Gierliński, 2011). Well-separated phalangeal pads are clearly visible in at least 2 tracks attributed to morphotype B (Figs. 5B, 7B, Figs. S7F–S7G). Furthermore, the CPR of *Therangospodus* (2.91) does not match the mean of morphotype B (4.30). And *Therangospodus* is typically a Late Jurassic ichnogenus, while these tracks are early Jurassic in age (Lockley, Meyer & Moratalla, 1998; Xing, Harris & Gierliński, 2011). Based on the plots (Fig. 6) and medium size, several ichnospecies of *Kayentapus* show affinities with morphotype B, but one is particularly similar: *K. xiaohebaensis*, from the Fengjiahe Formation (Zhen et al., 1986). The size (28 cm), PR and CPR (2.20 and 5.01), L/W ratio (1.2) and divarication angle of outer digits (75°) are consistent (Fig. 7E). The original diagnosis describes *K. xiaohebaensis* as being biped, digitigrade, with three clawed toes (II, III, IV), no impression of hallux nor toe V, and no tail impression. The angles between the phalanges are large: II-30°-III−45°-IV, phalangeal pads are elliptic, and the crease is wide (Zhen et al., 1986). On this basis, and despite the poor preservation of most of morphotype B tracks, we have sufficient evidence to refer these tracks to *K. xiaohebaensis.*

The track referred to morphotype C is large. Based on the above-mentioned description, it does not seem to match one of the ichnospecies already reported from the Fengjiahe Formation, but it is reminiscent of some *Eubrontes* and *Kayentapus* specimens.

The track of morphotype C show affinities with the ichnogenus *Eubrontes*, including in the plots (Fig. 6), and especially regarding the size range. It is particularly close in proportions to *E. glenrosensis* from Nei Mongol (Li et al., 2010) but the latter appears to be generally larger with robust digits, while morphotype C has slender digits. The large size, projection ratio, low L/W ratio as well as the high divarication angle IIˆIV seem to indicate a *Kayentapus*-like track. *Kayentapus* is an ichnogenus with large tridactyl footprints (average pes length of 35 cm approximately), originally described by Welles (1971), based on a trackway from the Lower Jurassic Kayenta Formation of Arizona. It is characterized by the following features: track length ranging from 15 cm to 40 cm (Lockley, 2000); high divarication angle IIˆIV; divarication angle IIIˆIV higher than IIˆIII; well defined metatarsophalangeal pad behind digit IV (Welles, 1971; Piubelli, Avanzini & Mietto, 2005). However, as noted by Lockley, Gierliński & Lucas (2011), Welles (1971) did not originally discuss preservation. It is therefore possible that some of the distinctive features result from differential preservation. In which case, the lack of a metatarsophalangeal impression sometimes observed in *Kayentapus* may be an extra morphological feature. The closest footprints to morphotype C in terms of morphology are *K. hailiutuensis* from Nei Mongol (Li et al., 2010), *K. wumaensis* from Sichuan (Yang & Yang, 1987), and *K. hopii* from Chongqing city (Xing et al., 2020). *K. hailiutuensis* is smaller in size, with a higher divarication angle of outer digits (83°) and a rather unspecific diagnosis (Li et al., 2010). The state of preservation makes the morphology, and interpretative drawing, ambiguous (Li et al., 2010, p. 740): either the digits are very robust, or they are very slender with digits II and IV very short relative to digit III. In both cases, it does not match morphotype C. *K. wumaensis* is 30 cm in length, and has consistent L/W and PR, but “nail”-like digits, which are not observed in morphotype C. Here too, the available illustration is not very informative and the diagnosis rather unspecific (Yang & Yang, 1987). *K. hopii* from the Early Jurassic of Chongqing city appears to be the closest form to morphotype C (Fig. 7F). The ichnofossils described by Xing et al. (2020) consist of an assemblage of 44 tracks, most of which were referred to *K. hopii*. The average divarication is consistent with morphotype C, but the tracks are, on average, smaller in size (24 cm), with a slightly lower average PR (1.4) and a slightly higher average L/W ratio (1.0). We could propose to refer morphotype C to *K. hopii* if it was not for the original diagnosis of the ichnogenus stating that there is an isolated metatarsal pad (Welles, 1971). This feature is not observed in morphotype C. Subsequently, morphotype C cannot be reliably referred to an existing ichnospecies of *Kayentapus* and, lacking diagnostic characters of its own, is therefore identified as *Kayentapus isp*.

The above taxonomic attributions might not be definitive considering the current state of affairs of dinosaur paleoichnology, especially in China (Lucas, 2001; Lockley et al., 2013). Moreover, as argued by Gierliński (1994) and reiterated by Lockley, Matsukawa & Li (2003), Lü et al. (2007b), Lockley & Matsukawa (2009) and Lockley et al. (2013), large theropod tracks from the Early to Middle Jurassic of China cannot be differentiated from *Eubrontes* or *Kayentapus* without relying on small, qualitative features, such as divarication angles that can be impacted by pes-substrate interactions or trackmaker behavior (Demathieu, 1990; Milàn, 2006; Li, 2015). Still, vertebrate footprint ichnotaxonomy is mostly based on track morphology (Marchetti et al., 2019) which, in turn, is determined by three factors: anatomy of the trackmaker’s foot, substrate properties, and behavior (Jackson, Whyte & Romano, 2010; Falkingham, 2014). Additional factors affecting track morphology may also include pre-burial and/or taphonomic alterations (Henderson, 2006; Scott, Renaut & Owen, 2010), as well as diagenesis (Lockley, 1999; Schulp, 2002; Lockley & Xing, 2015). Xiyang track site provides an excellent example for the track variation due to extra morphological factors as tracks from the same layer show differential preservation: the general shape, as well as the pad or claw impressions can be affected. For instance, tracks XIY-065 to 076 (morphotypes A and B) are all on the same layer, but XIY-065 (morphotype A) is deeper and more distinct, while others appear to be shallower and less defined. A similar phenomenon is also witnessed on other layers. Such variation could be due to the time span between the visits of trackmakers and therefore, substrate consistency. Even the footprints left by the same individual can present slightly different morphologies (Gatesy et al., 1999; Milàn, 2006; Razzolini et al., 2014; Razzolini et al., 2016), as observed in TA1 (Fig. S9).

### Trackmaker identity, size and speed

Olsen, Smith & McDonald (1998) noted the global similarity between the reconstructed osteology of some theropod footprints and early Mesozoic basal sauropodomorph or ornithischian feet. By plotting data about footprint proportions, they demonstrated that different fields appear for theropods, basal sauropodomorphs and ornithischians, and that there is not much overlapping (Farlow & Lockley, 1993; Olsen, Smith & McDonald, 1998: fig. 16). In the same vein, and on multiple occasions, this point was emphasized by Weems (1992), Weems (2003), Weems (2006b) and, more recently, Weems (2019). Through a thorough analysis of tracks (hallux impressions, foot proportions, pes musculature), as well as their abundance and stratigraphic range, Weems (2019) demonstrated that some *Eubrontes* and *Kayentapus* footprints reported in China (e.g., *E. zigongensis*, *K. nananensis*) were left by basal sauropodomorphs rather than theropods. The sets of tracks we reported from Xiyang track site are not affected by these conclusions, as none of them has proportions consistent with *Eubrontes*, nor the hallux impression diagnostic of *K. nananensis*. Basal sauropodomorph footprints were not observed *in situ* and, interestingly enough, were never reported in Yunnan either whilst the Province has yielded abundant body fossils of this group (Young, 1941; Young, 1942; Young, 1947; Simmons, 1965; Yang, 1982; Chao, 1985; Bai, Yang & Wang, 1990; Dong, 1992; Zhang & Yang, 1995; Fang et al., 2000; Fang et al., 2004; Lü et al., 2006; Lü et al., 2007a; Lü et al., 2008; Upchurch et al., 2007; Sekiya, 2010; Xing et al., 2015; Wang et al., 2017; Zhang et al., 2018). It is very puzzling because the well-known Lufeng Saurischian Fauna is dominated by basal sauropodomorphs while theropod body fossils are scarce, and basal sauropodomorphs were the dominant group in Early Jurassic of Yunnan. A potential reason to explain this absence throughout Yunnan could be a sampling bias. Just as all the layers and footprints are not fully revealed in Xiyang track site, the same situation may occur in other localities. Alternatively, identification errors could be the cause, as it has been demonstrated that basal sauropodomorph tracks were often mistaken for theropod ones (Weems, 1992; Weems, 2003; Weems, 2006b; Weems, 2019). Ultimately, ecological niches and habits could also be a key factor, as herbivores usually perceive water points as risky habitats due to greater predation risk, and would visit them only furtively when it is absolutely necessary (Valeix et al., 2010). These assumptions will need to be tested and require more detailed work, as well as discovery of more ichnofossils in the future.

A total of six theropod species were unearthed from lower Jurassic deposits in Yunnan, namely: *Eshanosaurus deguchiianus* (Xu, Zhao & Clark, 2001), *Lukousaurus yini* (Young, 1940), which might be a crurotarsan (see Irmis, 2004), *Megapnosaurus sp*. (Irmis, 2004), *Panguraptor lufengensis* (You et al., 2014), *Shuangbaisaurus anlongbaoensis* (Wang et al., 2017) and *Sinosaurus triassicus* (formerly *Dilophosaurus sinensis*) (Young, 1948; Hu, 1993). In the Fengjiahe Formation where the Xiyang track site lies, three theropod genera are known: *Sinosaurus, Eshanosaurus*, and *Shuangbaisaurus* (Hu, 1993; Xu, Zhao & Clark, 2001; Wang et al., 2017). Interestingly, a subcomplete specimen of *Sinosaurus* (Hu, 1993) was excavated nearby the Xiyang track site geographically (Fig. 1), and *Eshanosaurus* (Xu, Zhao & Clark, 2001) was also collected in this area. Based on the identified morphotypes, we believe that the Xiyang track site records the movements of two or three theropod species. The morphotype A trackmaker has an average calculated hip height of 0.7 m / 0.6 m (using Thulborn, 1990 and Alexander, 1976 methods, respectively) and an average length of 2.6 m / 2.3 m (Table S5). As mentioned above, the Fengjiahe Formation shares a similar fauna with its equivalent, the Lufeng Formation. Thus, the *Grallator*-like (morphotype A) trackmaker is most probably the small *Panguraptor*, which body length is estimated around 2 m (You et al., 2014), or a closely related coelophysoid theropod. *Eshanosaurus*, a putative therizinosaur only known from an isolated dentary, is possibly in the size range as well (Xu, Zhao & Clark, 2001; Barrett, 2009). Consequently, it could also be responsible for the tracks referred to morphotype A. However, therizinosaurs are one of two groups of theropods known to have four forward-facing digits (Fiorillo & Adams, 2012), so their morphology does not match the footprints seen here. Size estimation for the *Kayentapus xiaohebaensis* (morphotype B) trackmaker is 1.4 m / 1.4 m at hip height and 5.5 m / 4.5 m long on average (Table S5). The *Kayentapus isp.* (morphotype C) trackmaker, in turn, has a 1.9 m / 1.6 m hip height and would be 7.3 m / 6.6 m long (Table S5). Morphotypes B and C correspond in all likelihood to larger, tetanuran, theropods. *Sinosaurus* and *Shuangbaisaurus*, from the Fengjiahe Formation, have body lengths estimated around 5–6 m (Hu, 1993; Wang et al., 2017) and could therefore be the trackmakers. Incidentally, the fact that these two morphotypes are referred to the same ichnogenus (*Kayentapus*) could also imply ontogeny.

Co-occurrence of tracks of similar morphology but significantly different sizes or digit divarication is observed in morphotype A, as it was also observed in other theropod track sites from Xinjiang Province (Xing et al., 2014a). Following this, morphotype A can be interpreted as having been left by individuals of the same species at different ontogenetic stages. The proximity of track association 1 and trackway 1 supports this hypothesis (Fig. S9). Their footprints share a similar morphology, impression, and direction of movement; the lateral divarication angle and PR, CPR are also close, the most distinct difference is size, which could supposedly be evidence of social behavior. This proximity may also be attributable to a gap in time between one passing and another passing. Repeated use of a resource area does not necessarily imply congregation. Given the small size of morphotype A footprints, another assumption could be that they were produced predominantly by juvenile individuals. However, no dominant size class (e.g., all tracks strictly around 10 cm long) or pattern (e.g., small tracks escorted by large ones, with a clear size gap) is identifiable within morphotype A (Fig. S14). Sizes follow a continuum, which means that one age or one species was not present in larger numbers. Alternatively, morphotype A could also have been produced by different species with identical pes morphology.

The speed analysis of the trackway, according to Alexander’s (1976) method, gives results estimated at 3 m/s and 3.6 m/s for the coelophysoid trackmaker (depending on which hip height formula is considered; see Table S5). SL/H being 2.9, we infer that the animal’s gait was trotting.

### Paleoenvironment

Following the work of Lockley (1991) and Lockley & Hunt (1996) on the categorization of formations according to the relative abundance of trace and body fossils, the Fengjiahe Formation appears to be type 4b (bone-dominated, with track and bone record being mostly inconsistent).

Based on the lithology and sedimentological structures, as well as the impression and depth of most footprints, we infer that the substrate was firm and probably drying-out when most of the animals came by. Still, some deep tracks such as XIY-048 were obviously left in a soaked substrate (the footprint is on a sandstone layer with ripple marks). Most tracks from Xiyang track site are preserved in silty claystone, some in sandstone. Mudcracks are the most common sedimentological structure found in association with footprints. According to our observations, ripple marks on the sandstone layer are symmetrical and this same layer is overlain by a bed with mudcracks, which suggests water level fluctuations. Based on the aforementioned information, the Xiyang track site experienced several drought events in a short time frame, which provided good conditions for the preservation of tracks on several layers (Lockley, 1991; Paik, Kim & Lee, 2001).

**Figure 8:**
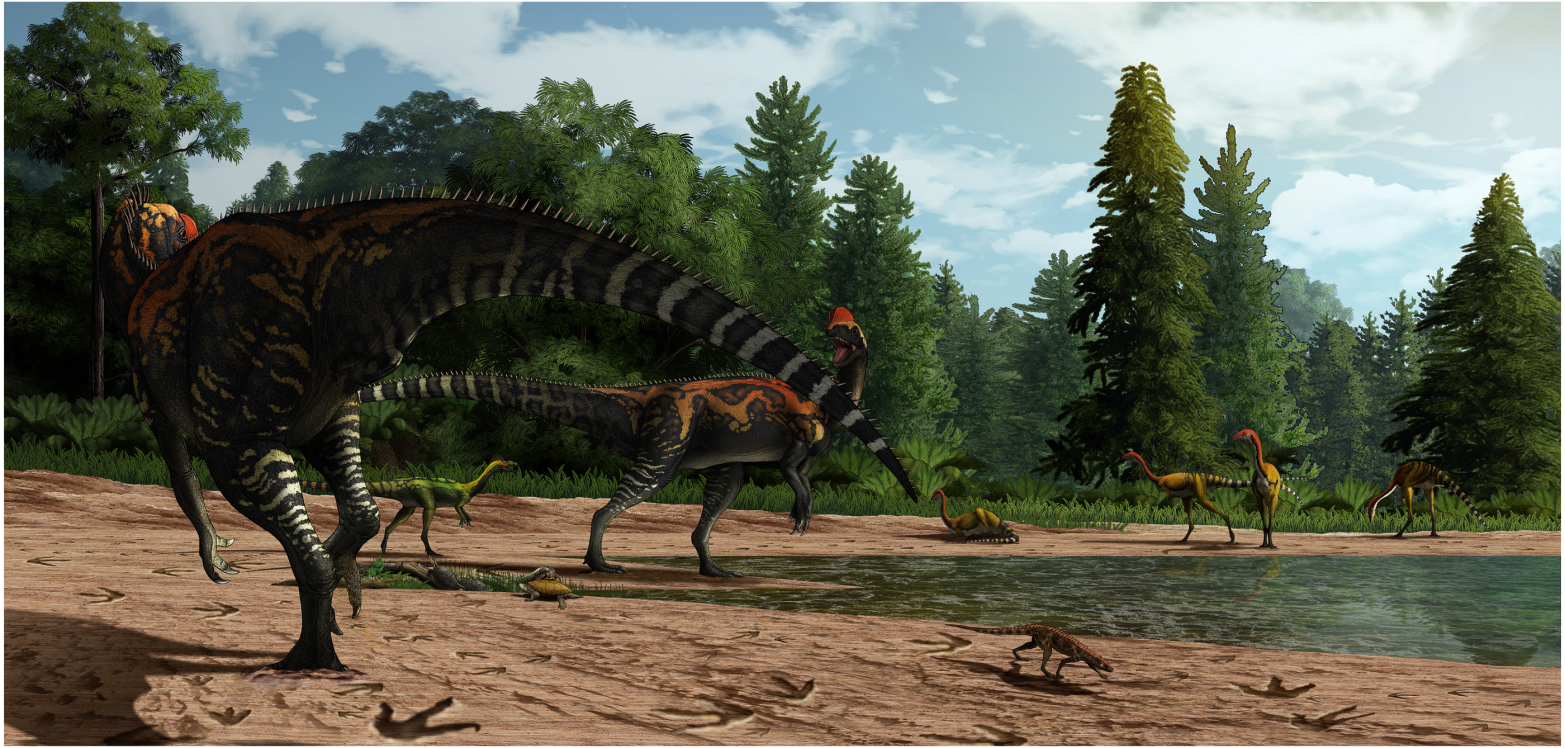
Paleoenvironmental reconstruction of the Xiyang track site by Yu Chen.

The depth difference and different preservation conditions among and the tracks reveal that most of them are not strictly contemporaneous. The occurrence of different layers and their lithologic composition support this claim as they demonstrate that the units were not deposited (and hence walked upon) simultaneously. Thus, we estimate that multiple generations of theropods have visited the site, most likely in a short time span. The morphological differences among these tracks imply that theropods of different sizes and types stopped by this site frequently, most likely attracted by food or water resources (Fig. 8; Cohen et al., 1993). However, the random arrangement of the footprints suggests limited interactions between trackmakers (Moreno et al., 2012). Tracks from the Xiyang track site suggest that theropods flourished in the Jurassic of central Yunnan, and add to the growing track record of the body fossils dominated Fengjiahe Formation.

## Conclusions

The Xiyang track site preserves 120 exposed footprints made by solitary coelophysoid and tetanuran theropod dinosaurs within a lacustrine setting under tropical paleoclimatic conditions. The footprints are grouped into three morphotypes and show similarities with two widespread ichnogenera: *Grallator* and *Kayentapus*. These ichnogenera were both already reported in the Fengjiahe Formation, but not in its lateral equivalent the Lufeng Formation.

The track site is dated from the Late Early Jurassic, and the locality is close to the Lower-Middle Jurassic boundary. In equivalent levels of the Lufeng Formation, typical components of the *Lufengosaurus* fauna are abundant. Hence, the Xiyang track site can be regarded as part of this fauna. Tracks preserved on multiple layers suggest that this area underwent periodic droughts and flood events. It also implies that dinosaurs of different generations and sizes kept visiting the site.

In the Fengjiahe Formation, the record is dominated by body fossils while ichnofossils are relatively limited. Curiously, little is known about tracks in the highly fossiliferous Yunnan Province. This might be due to the small number of previous studies and lack of suitable facies for abundant track preservation. The Xiyang track site is the track site with the greatest number of theropod footprints found and reported in Yunnan so far. Thus, it provides valuable insights into the diversity and ecology of Early Jurassic theropods in Yunnan. Because theropods are relatively sparse in Yunnan, and some genera were erected on basis of scattered specimens, tracks can help fill the gap to some extent.

##  Supplemental Information

10.7717/peerj.11788/supp-1 Supplemental Information 1 Supplemental Figures and Tables

10.7717/peerj.11788/supp-2 Supplemental Information 2 Measurements of the tridactyl tracks from the Xiyang track site

## Additional Information and Declarations

### Field Study Permissions

The following information was supplied relating to field study approvals (i.e., approving body and any reference numbers): Authorizations were given verbally by Mr Deke Zhao for the Culture and Tourism Bureau of Xiyang Township.

### Data Availability

The following information was supplied regarding data availability: The specimen is available at Morphosource (000360513): DOI 10.17602/M2/M360516.
