# Supplementary material for: The largest theropod track site in Yunnan, China: a footprint assemblage from the Lower Jurassic Fengjiahe Formation
Source: PeerJ. 2021 Oct 5;9:e11788. doi: 10.7717/peerj.11788 (PMC8500084; doi:10.7717/peerj.11788)
Supplement: Supplemental Information 1 [file peerj-09-11788-s001.pdf]

SUPPLEMENTARY INFORMATION

The largest theropod track site in Yunnan, China: a footprint assemblage from the Lower Jurassic Fengjiahe Formation

Hongqing Li[1], Claire Peyre de Fabrègues[1], Shundong Bi[2,1], Yi Wang[3], Xing Xu[4,5]

[1] Centre for Vertebrate Evolutionary Biology, Yunnan University, Kunming, Yunnan Province, China

[2] Department of Biology, Indiana University of Pennsylvania, Indiana, Pennsylvania, USA

[3] Yuxi Museum, Yuxi, Yunnan Province, China

[4] Key Laboratory of Vertebrate Evolution and Human Origins, Institute of Vertebrate Paleontology and Paleoanthropology, Chinese Academy of Sciences, Beijing, China

[5] Center for Excellence in Life and Paleoenvironment, Chinese Academy of Sciences, Beijing, China

Figure S1. Lower-Middle Jurassic boundary

Figure S2. Ripple marks

Figure S3. Overview of the track site with track numbers

Figure S4. Measurement guidelines

Figure S5. Interpretative outline drawing of all the tracks

Figure S6. Photographs of all the tracks

Figure S7. Photographs of some of the well-preserved tracks

Figure S8. False-color depth map of track XIY-048

Figure S9. Photograph and interpretative outline drawing of track association 1 & trackway 1

Figure S10. Photograph and interpretative outline drawing of track association 2

Figure S11. Photograph and interpretative outline drawing of track association 3

Figure S12. Photograph and interpretative outline drawing of track association 4

Figure S13. Photograph and interpretative outline drawing of track association 5

Figure S14. Graph of the lengths of all the most complete footprints

Table S1. Measurements of the tridactyl tracks from the Xiyang track site

Table S2. Preservation scale

Table S3. Specimen numbers and measurements of theropod ichnospecies holotypes from the Jurassic of China

Table S4. Comparison of morphotype A with theropod ichnospecies reported from the Fengjiahe Formation

Table S5. Calculated hip heights and body lengths for the 3 morphotypes

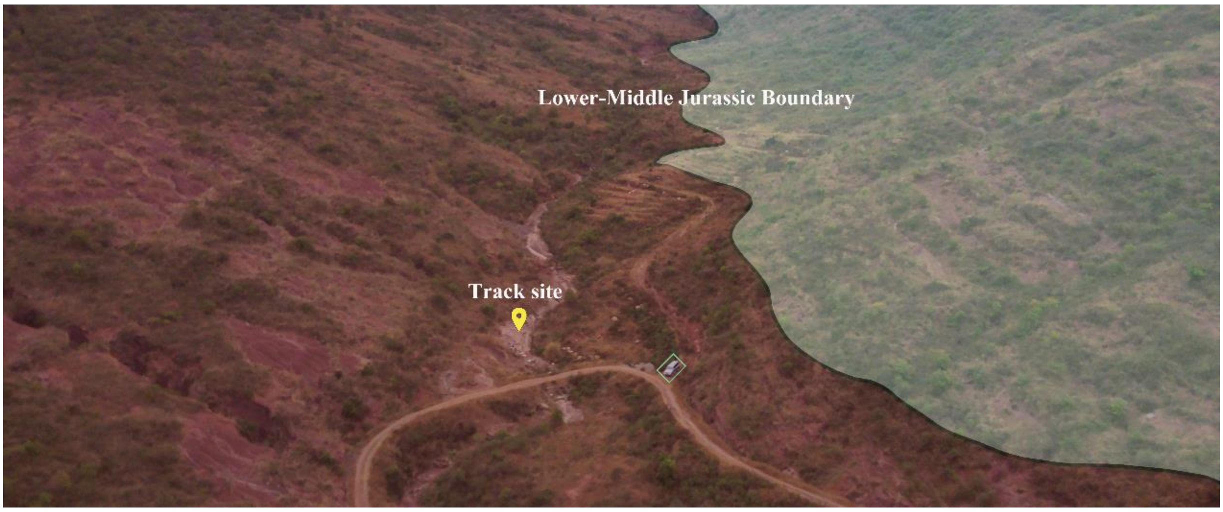

Figure S1. Lower-Middle Jurassic boundary relative to the Xiyang track site. The car in the green square indicates scale.

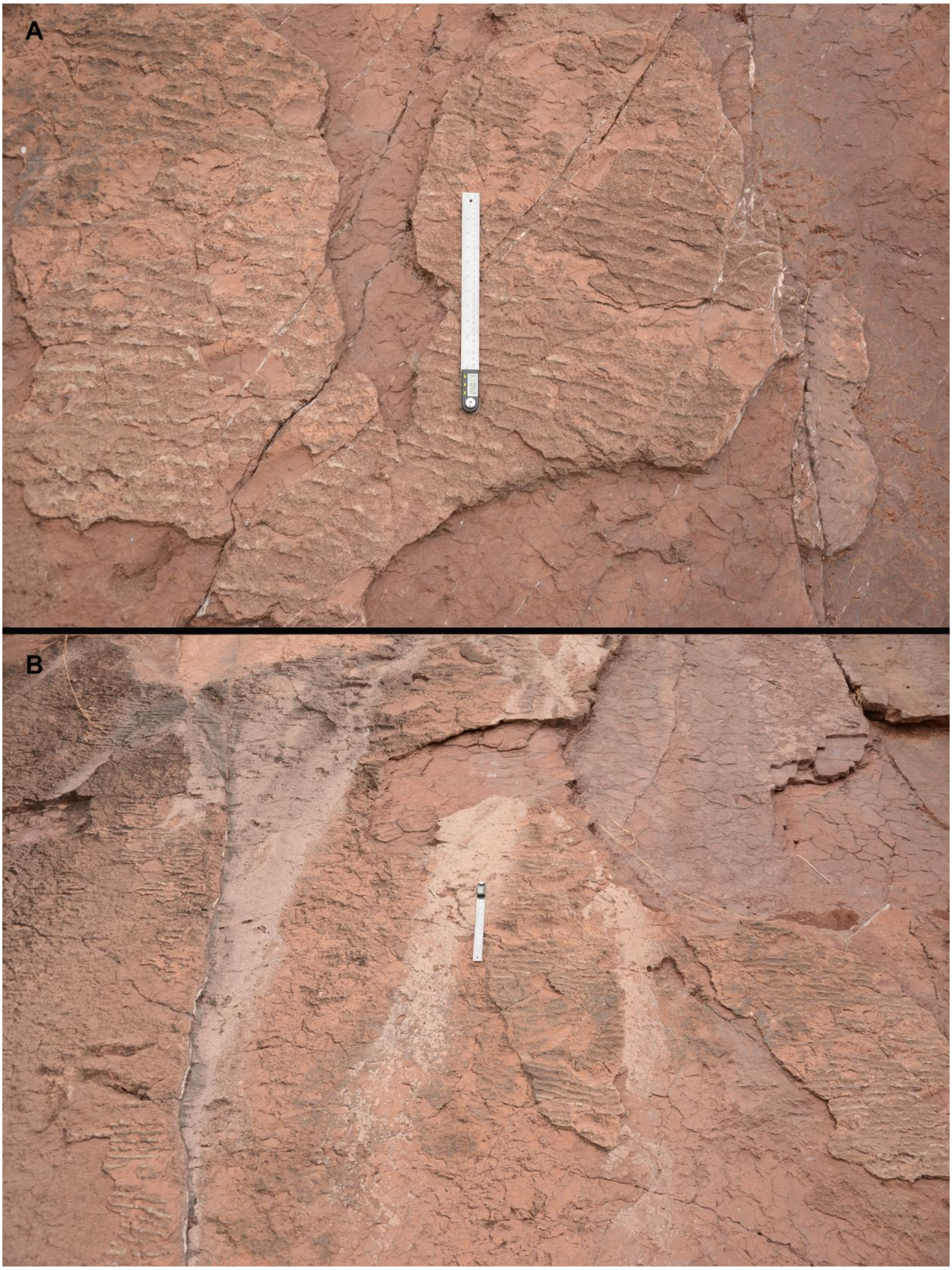

Figure S2. Ripple marks on the layers of the Xiyang track site. Scale bar is 30 cm.

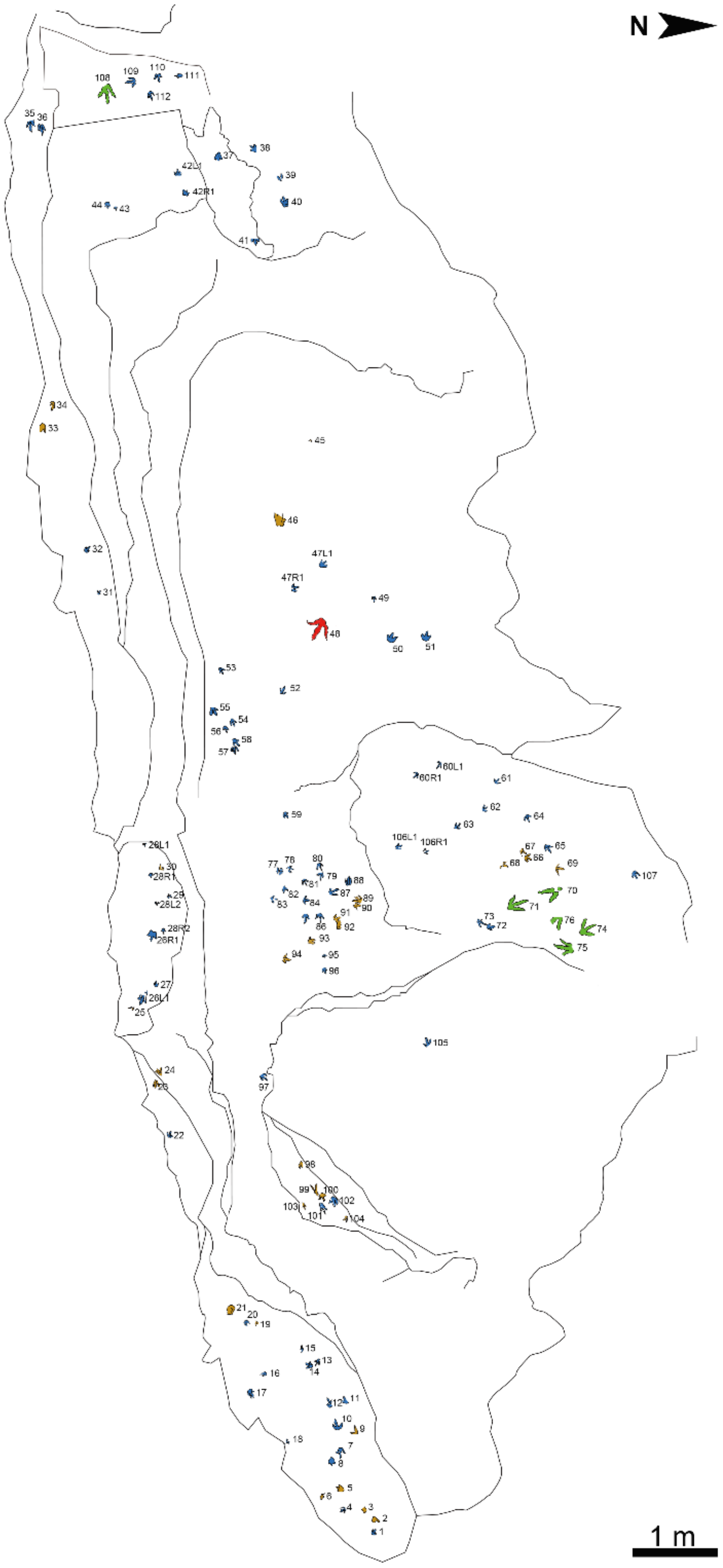

Figure S3. Overview of the Xiyang track site. Outline drawing showing the distribution and catalogue number of the tracks. Morphotypes A, B and C are painted in blue, green, and red, respectively; unidentified tracks appear in brown.

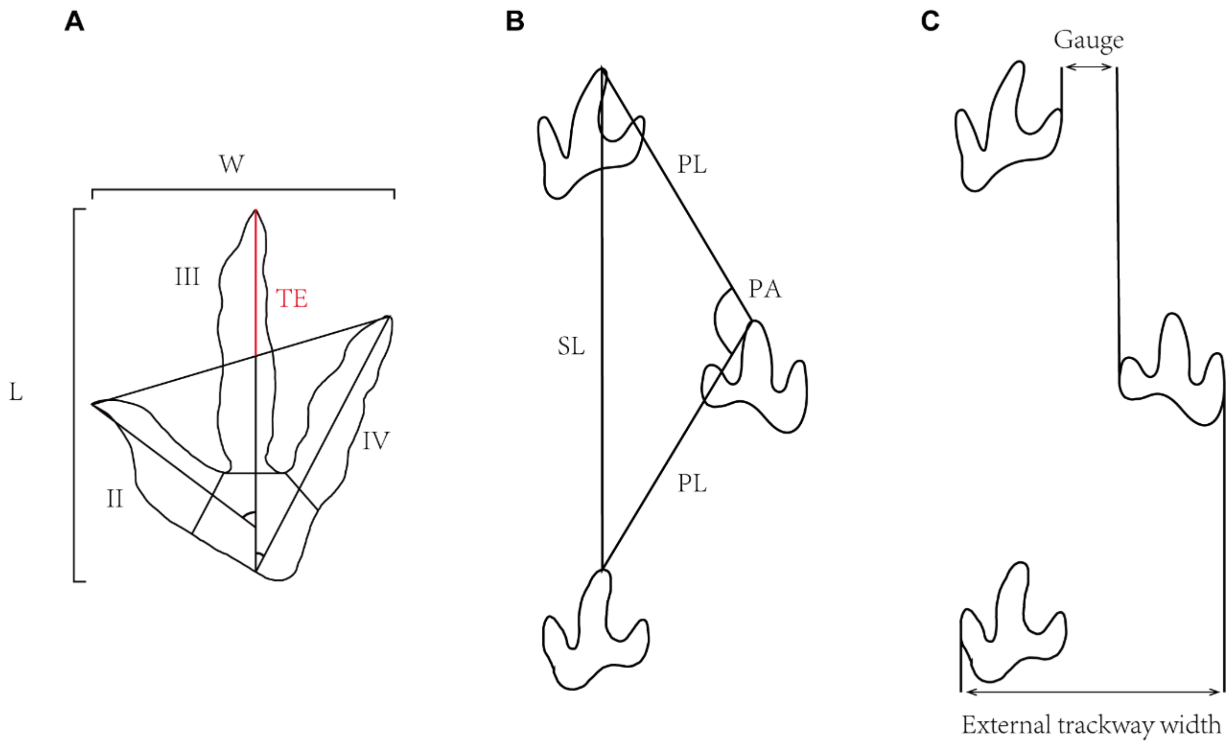

Figure S4. Measurement guidelines. (A) Measurements for isolated tracks. The track displayed is XIY-071(Morphotype B). (B-C) Measurements for trackways and track associations. Drawings from Thulborn (1990).

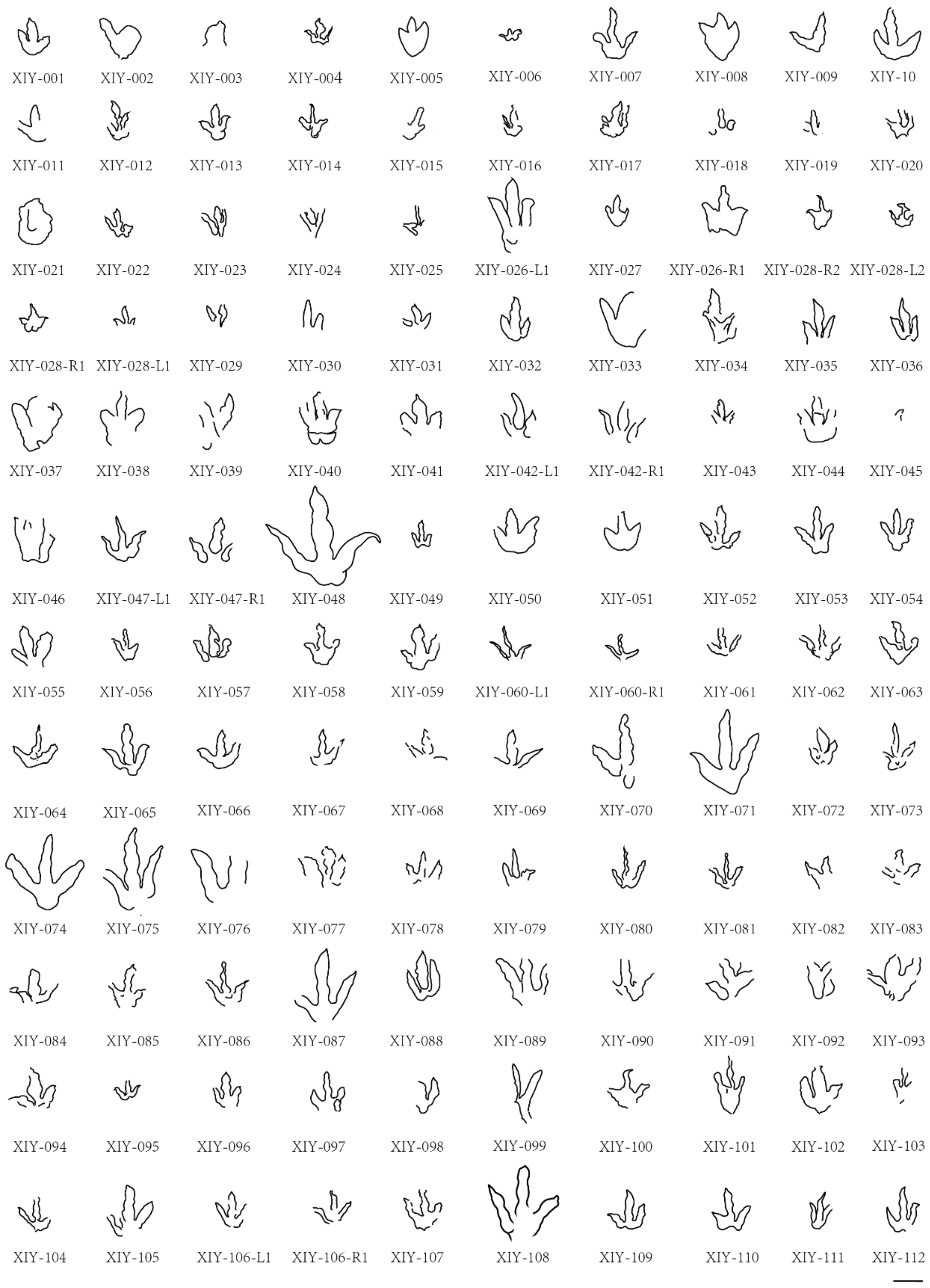

Figure S5. Interpretative outline drawings of all tridactyl dinosaur tracks from the Xiyang track site. Scale bar is 10 cm.

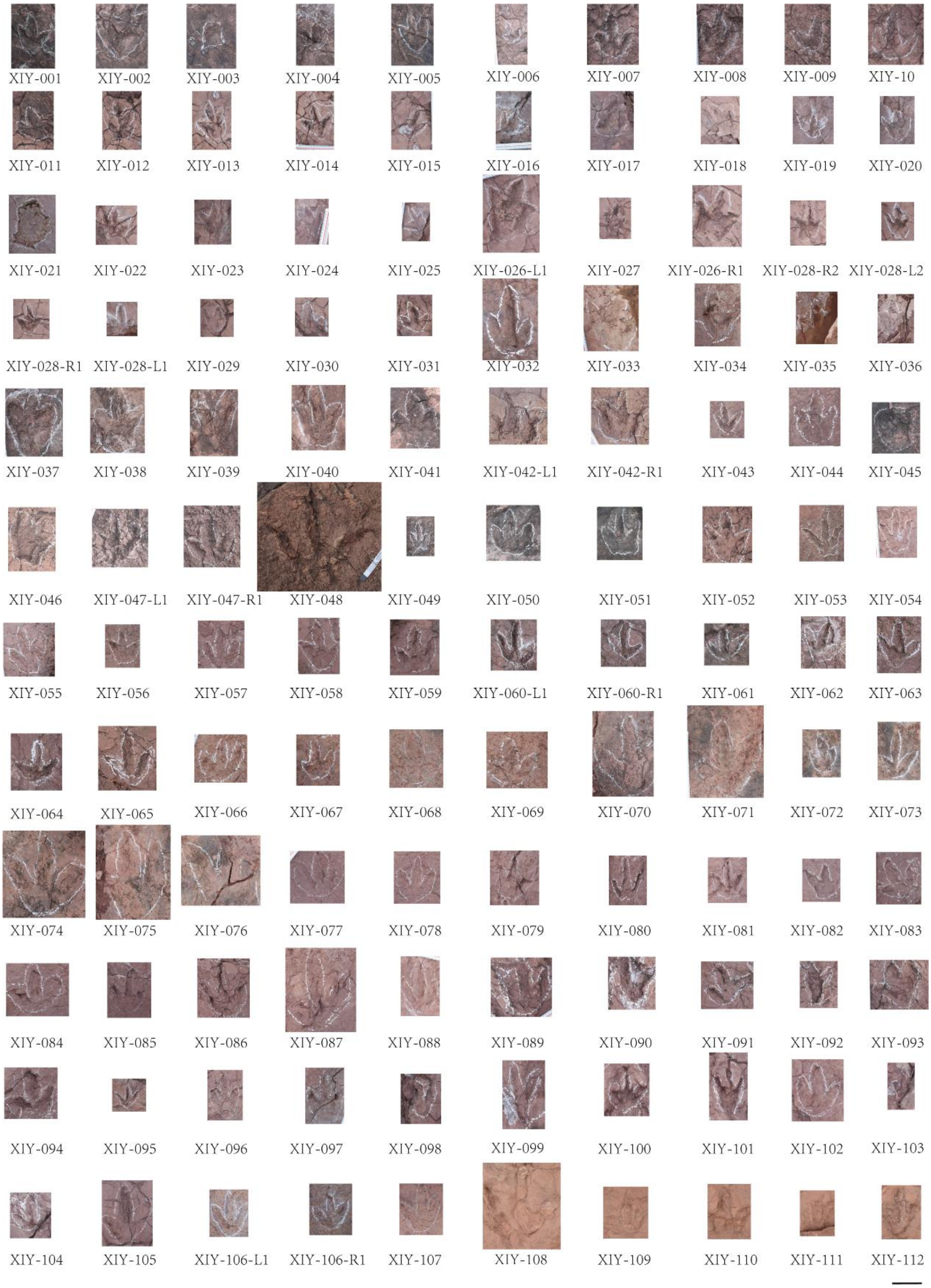

Figure S6. Photographs of all tridactyl dinosaur tracks from the Xiyang track site. Scale bar is 10 cm.

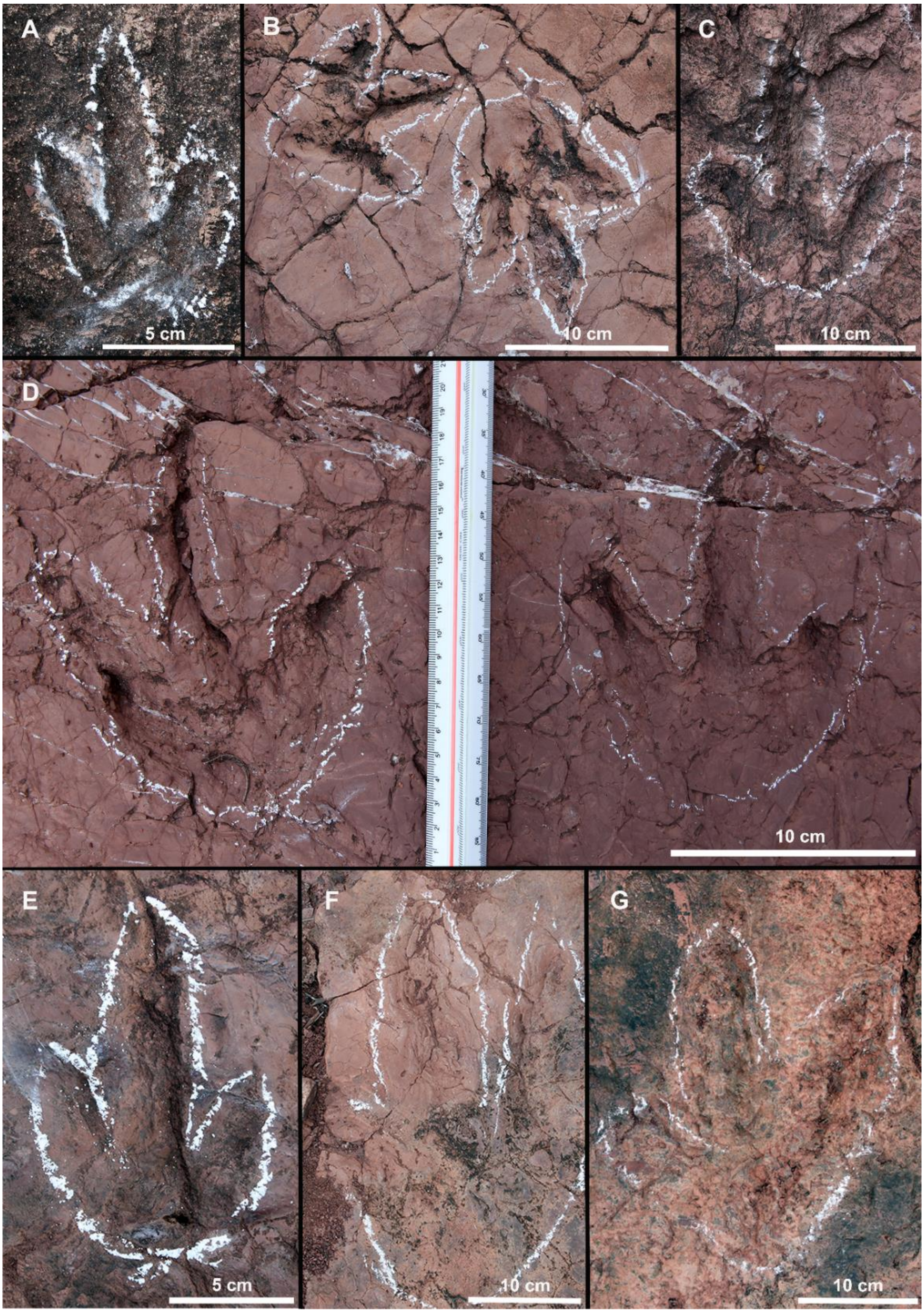

Figure S7. Photographs of some of the well-preserved tracks from the Xiyang track site. **(**A-E) Tracks referred to morphotype A (from A to E: XIY-049, 013 & 014, 007, 085 & 086, 032). (F-G) Tracks referred to morphotype B (XIY-075 and 071).

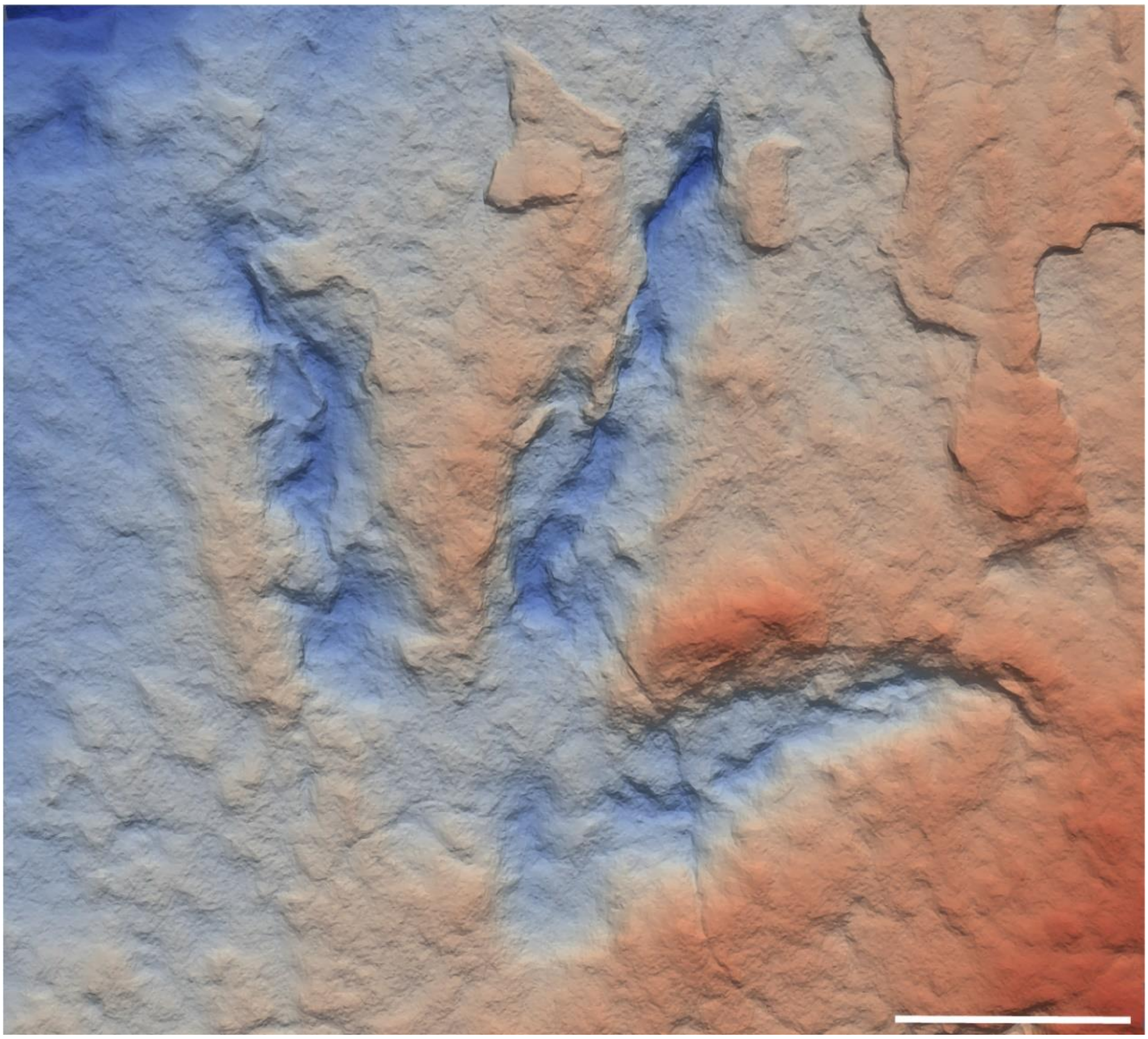

Figure S8. False-color depth map of track XIY-048 (morphotype C). Scale bar is 10 cm.

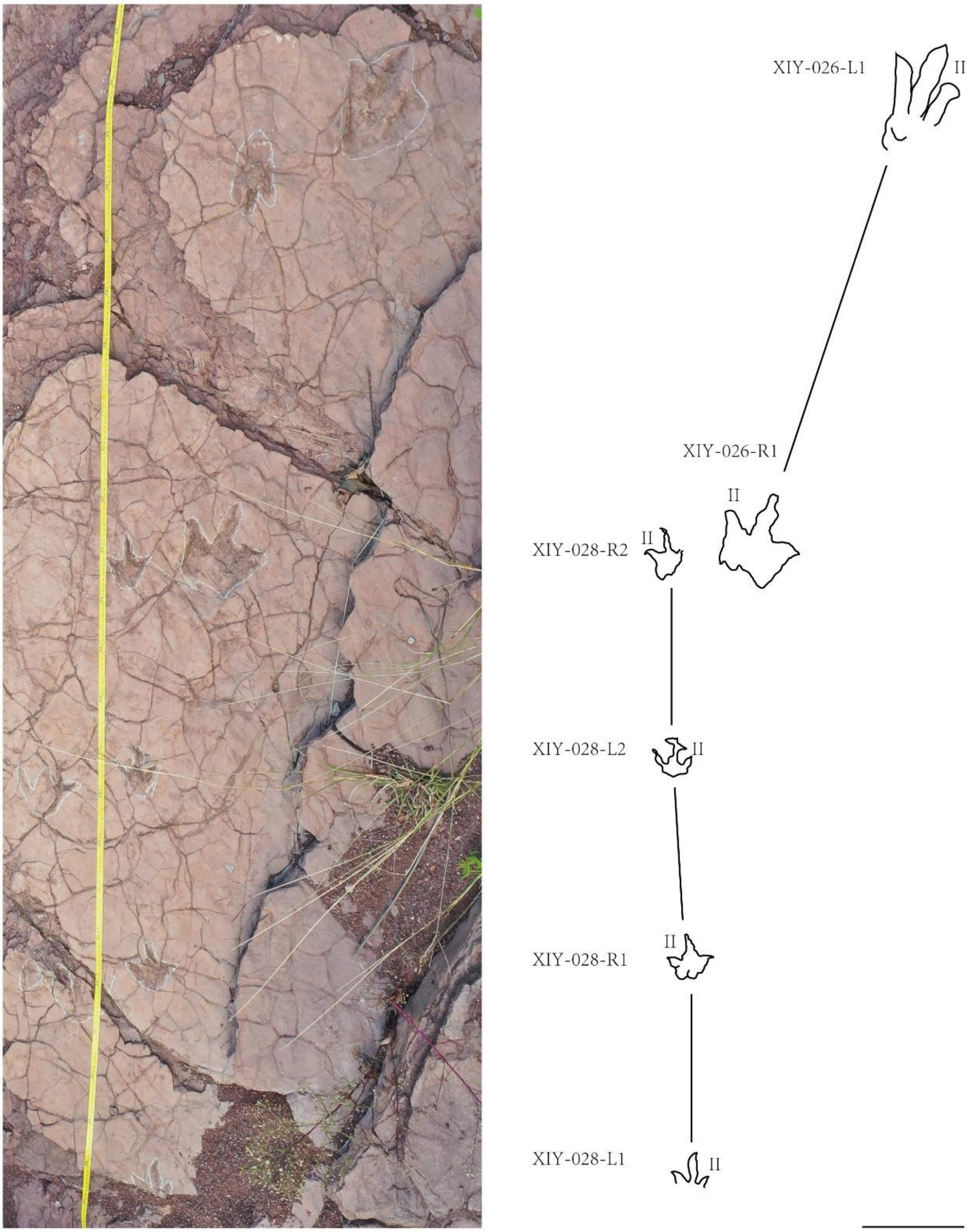

Figure S9. Photograph and interpretative outline drawing of track association 1 (XIY-026-R1 & L1) & trackway 1 (XIY-028-L1, R1, L2, & R2). Scale bar is 20 cm. Abbreviations: L= left pes, R= right pes.

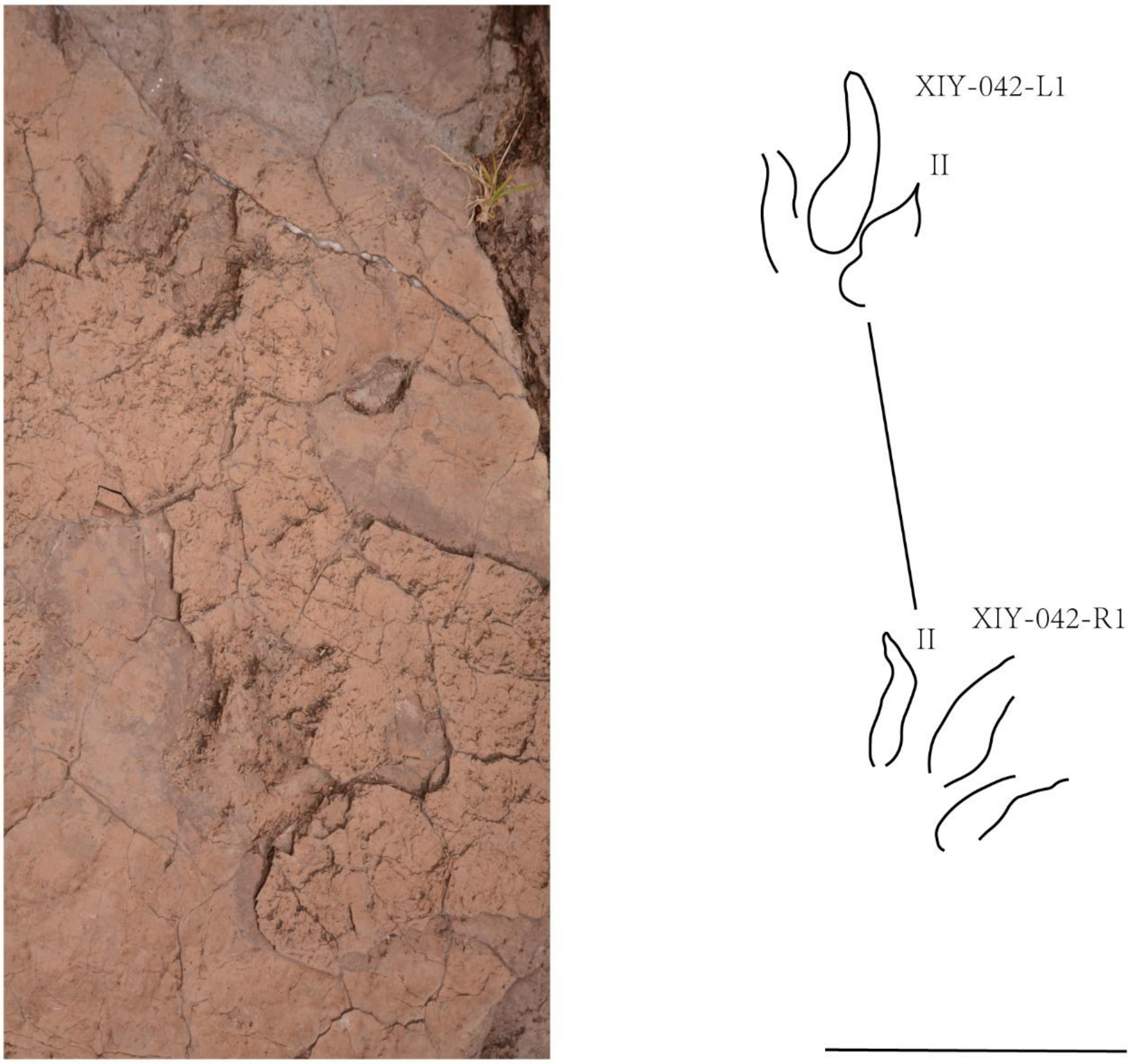

Figure S10. Photograph and interpretative outline drawing of track association 2 (XIY-042-R1 & L1). Scale bar is 20 cm. Abbreviations: L= left pes, R= right pes.

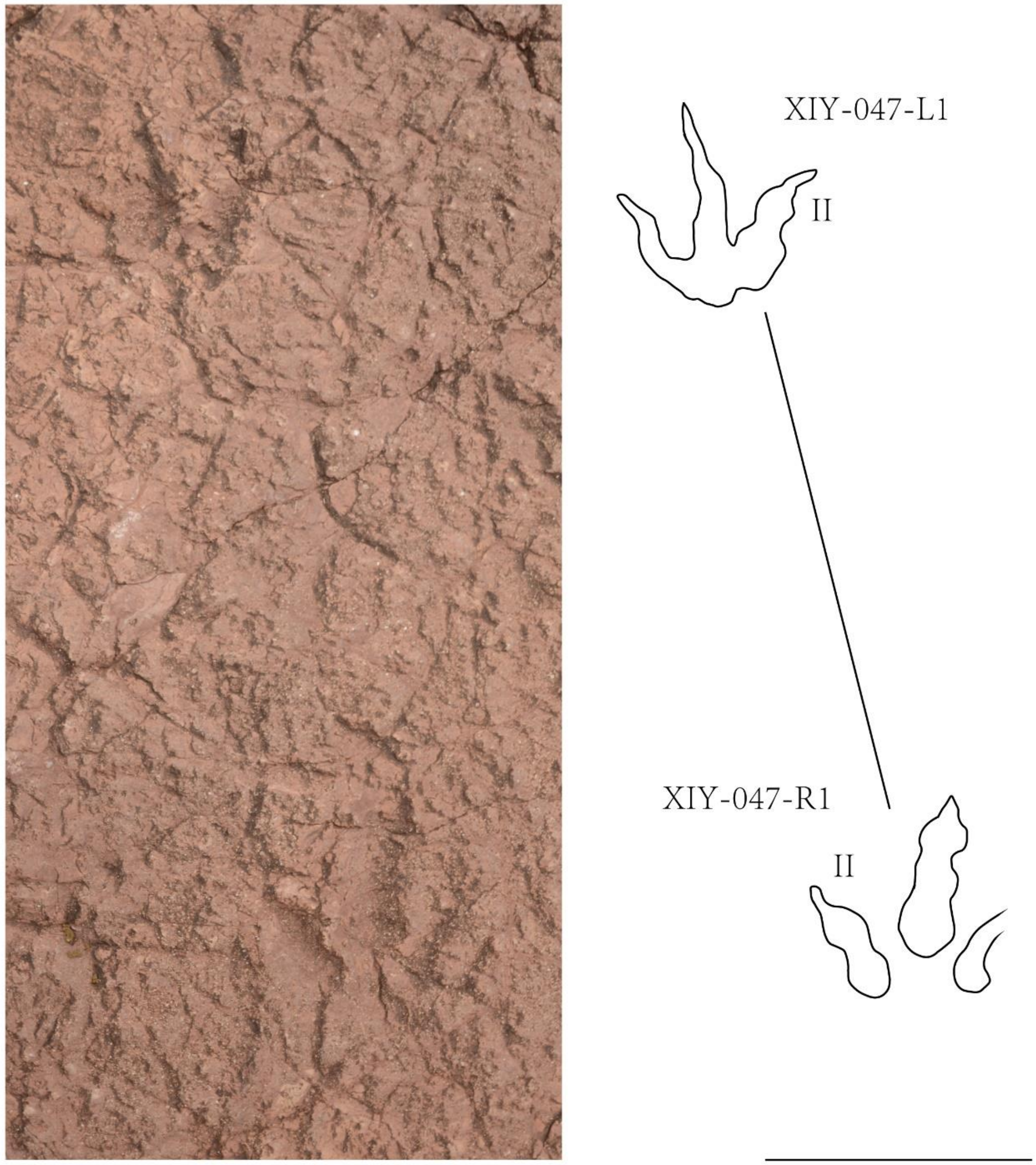

Figure S11. Photograph and interpretative outline drawing of track association 3 (XIY-047-R1 & L1). Scale bar is 20 cm. Abbreviations: L= left pes, R= right pes.

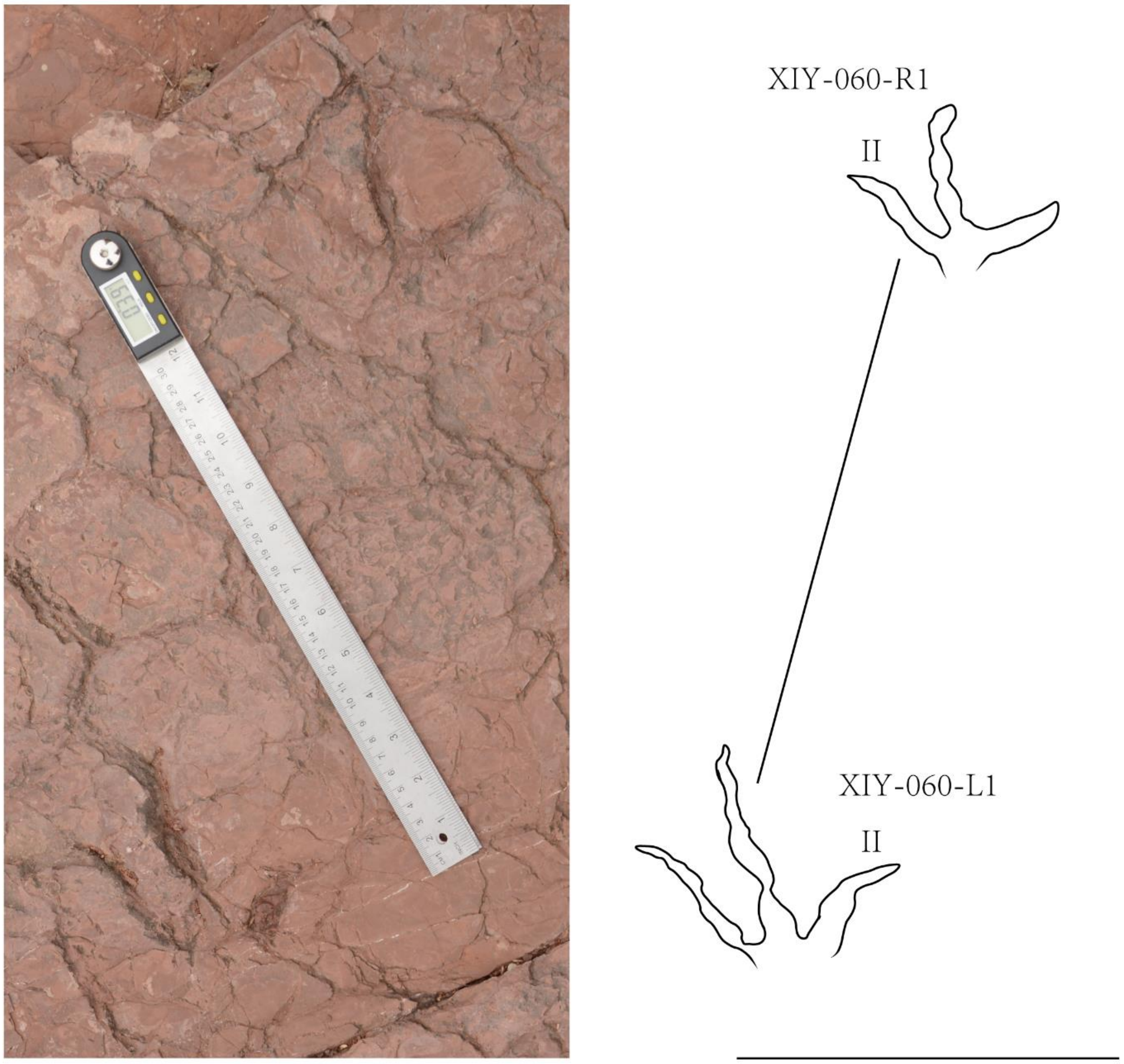

Figure S12. Photograph and interpretative outline drawing of track association 4 (XIY-060-L1 & R1). Scale bar is 20 cm. Abbreviations: L= left pes, R= right pes.

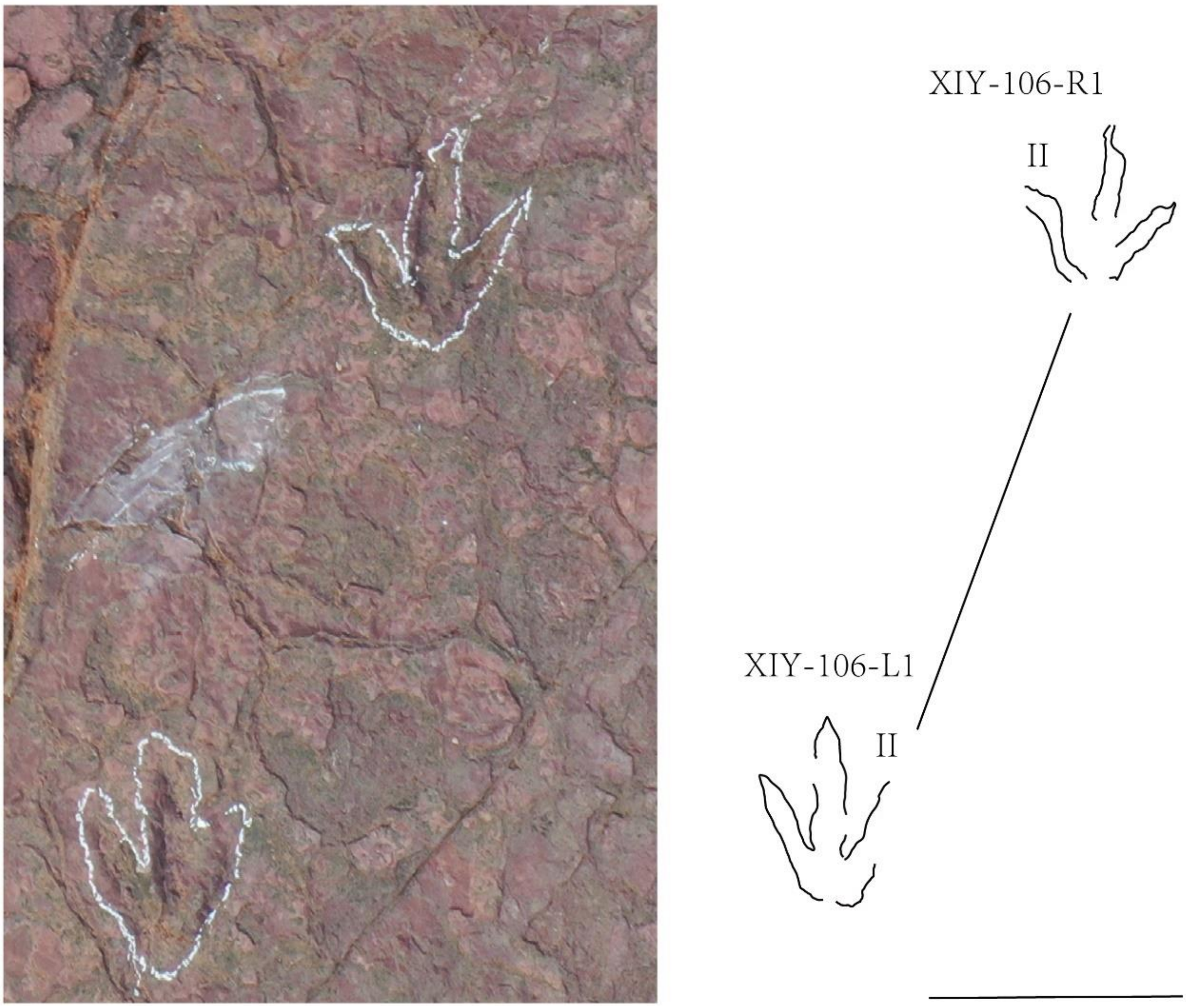

Figure S13. Photograph and interpretative outline drawing of track association 5 (XIY-106-L1 & R1). Scale bar is 20 cm. Abbreviations: L= left pes, R= right pes.

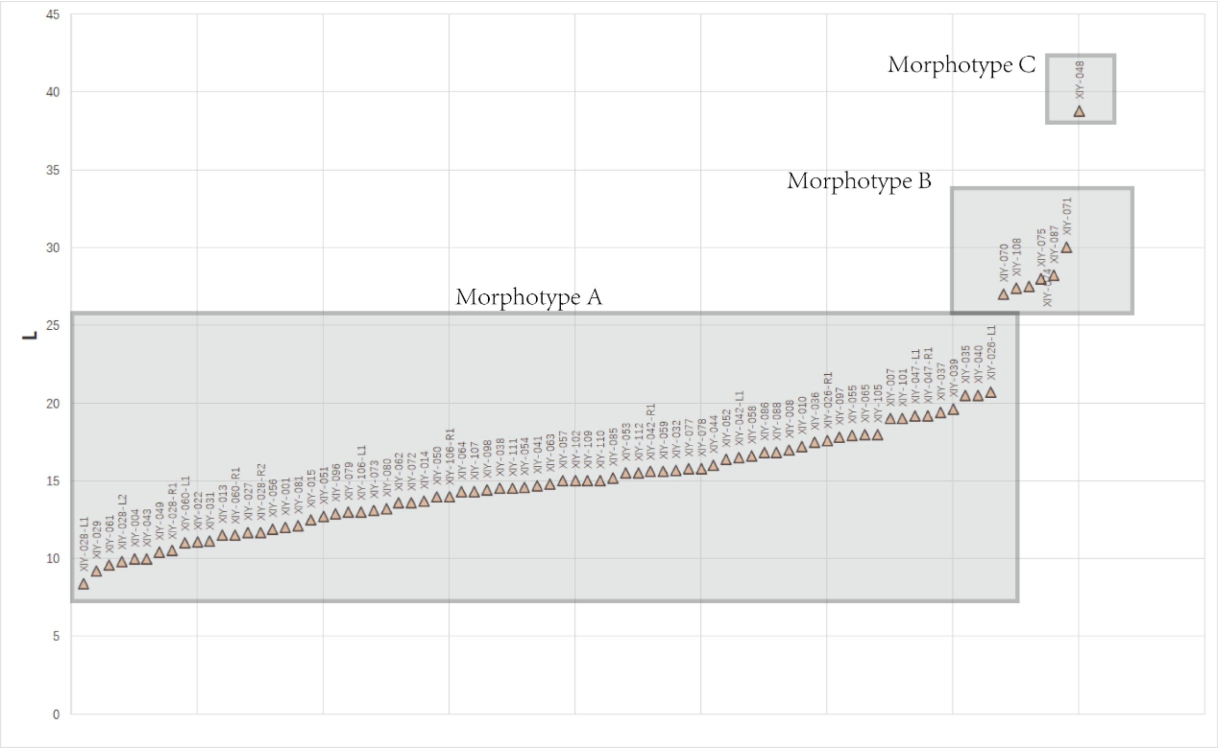

Figure S14. Graph of the lengths of all the most complete footprints. Tracks assigned to morphotype A are forming a continuum and clear gaps are visible between each morphotype.

Table S1. Measurements of the tridactyl tracks from the Xiyang track site. Abbreviations following the order of the table: M: Morphotype; L: maximum length (cm); W: maximum width (cm); LD: length of digit (cm); II-III to II-IV: angle between respective digits (degrees); PL: pace length (cm); SL: stride length (cm); PA: pace angulation (cm); L/W: length over width ratio; PR: projection ratio following Li, 2015 (p. 9); CPR: "corrected" projection ratio following Olsen, Smith & McDonald, 1998 (p. 586); LR: layer; *: incomplete; -: unavailable.

Number	M	L	W	LD II	LD III	LD IV	II-III	III-IV	II-IV	PL	SL	PA	L/W	PR	CPR	LR
**XIY-002**	-	-	14*	-	-	-	-	-	-	-	-	-	-	-	-	4
**XIY-003**	-	-	-	-	-	-	-	-	-	-	-	-	-	-	-	4
**XIY-005**	-	-	10.5	-	5*	-	-	-	-	-	-	-	-	-	-	4
**XIY-006**	-	-	7.6	-	-	-	-	-	-	-	-	-	-	-	-	4
**XIY-009**	-	12.5*	-	-	-	-	-	-	-	-	-	-	-	-	-	4
**XIY-011**	-	12.8	-	-	6.8	6.4*	-	-	-	-	-	-	-	-	-	4
**XIY-012**	-	-	-	-	-	-	-	-	-	-	-	-	-	-	-	4
**XIY-016**	-	-	-	-	-	-	-	-	-	-	-	-	-	-	-	4
**XIY-019**	-	-	-	-	6.2*	-	57*	-	-	-	-	-	-	-	-	4
**XIY-021**	-	-	-	-	-	-	-	-	-	-	-	-	-	-	-	4
**XIY-023**	-	-	9.8*	-	-	-	-	-	-	-	-	-	-	-	-	4
**XIY-024**	-	10.1	-	-	5.3*	-	-	-	-	-	-	-	-	-	-	4
**XIY-025**	-	9.8*	-	3.6	4.7*	-	56	-	-	-	-	-	-	-	-	4
**XIY-030**	-	10.7*	-	-	9.9*	-	-	-	-	-	-	-	-	-	-	4
**XIY-033**	-	19*	-	-	-	10*	-	-	-	-	-	-	-	-	-	4
**XIY-034**	-	21	-	-	11.3	8.9*	-	35	-	-	-	-	-	-	-	4
**XIY-045**	-	-	-	-	-	-	-	-	-	-	-	-	-	-	-	3
**XIY-046**	-	17	-	-	-	-	-	-	-	-	-	-	-	-	-	2
**XIY-066**	-	-	13.9	7.6	8.6*	8.3	56	21	77	-	-	-	-	-	-	2
**XIY-067**	-	12.6	-	5.6	7.7	-	33	39	72	-	-	-	-	-	-	2
**XIY-068**	-	-	-	-	-	-	30	30	60	-	-	-	-	-	-	2
**XIY-069**	-	-	16*	-	-	-	47*	29*	76*	-	-	-	-	-	-	2
**XIY-082**	-	11*	-	7.1	7.7*	-	38	-	-	-	-	-	-	-	-	2
**XIY-083**	-	12.2*	-	4.8	7.9*	5.6	-	-	-	-	-	-	-	-	-	2
**XIY-084**	-	12.7*	-	6.4	9.2	-	26	24	50	-	-	-	-	-	-	2

**XIY-089**	-	-	10.8*	7.74	-	9.2	19	19	38	-	-	-	-	-	-	2
**XIY-090**	-	-	16*	9.2	-	11.2*	23	17	40	-	-	-	-	-	-	2
**XIY-091**	-	-	-	-	-	-	-	-	-	-	-	-	-	-	-	2
**XIY-092**	-	13.1	-	5	8.6	-	23	-	-	-	-	-	-	-	-	2
**XIY-093**	-	16.2*	-	9	8.2*	-	34	26	60	-	-	-	-	-	-	2
**XIY-094**	-	-	17.9	8.5	11.2	7.1*	39	32	71	-	-	-	-	-	-	2
**XIY-099**	-	19.8*	-	-	10.2*	8.9*	-	31	-	-	-	-	-	-	-	2
**XIY-100**	-	-	13*	-	9.6*	-	22	34	56	-	-	-	-	-	-	2
**XIY-103**	-	10.2*	-	4.3	7	-	22	-	-	-	-	-	-	-	-	2
**XIY-104**	-	-	-	-	-	-	-	-	-	-	-	-	-	-	-	2
**XIY-001**	A	12	10	4.5	8.2	5.8	37	25	62	-	-	-	1.2	1.6	1.5	4
**XIY-004**	A	10	7.7	3.8	6.7	5	26	27	53	-	-	-	1.3	1.6	1.87	4
**XIY-007**	A	19	16	6.4	13.0*	8.8	24	27	51	-	-	-	1.19	1.2	1.31	4
**XIY-008**	A	17.0*	12	7.7	10.2	8.1	21	21	42	-	-	-	-	2.3	-	4
**XIY-010**	A	17.2	15.8	6.6	11.8	6.6	18	40	58	-	-	-	1.09	1.30	1.60	4
**XIY-013**	A	11.5	10.8	5.7	8.0	5.3	22	41	63	-	-	-	1.06	1.34	1.29	4
**XIY-014**	A	13.7	10.5	6.8	9.3	7.2	15	33	48	-	-	-	1.30	1.90	1.62	4
**XIY-015**	A	12.5	-	-	7.3	-	-	25	-	-	-	-	-	-	-	4
**XIY-017**	A	10.6*	9.6*	-	-	-	16	16	32	-	-	-	1.10	-	-	4
**XIY-018**	A	12	-	6.8*	6*	-	25	-	-	-	-	-	-	-	-	4
**XIY-020**	A	9.5	8.4	-	-	-	-	-	-	-	-	-	1.13	-	-	4
**XIY-022**	A	11.1*	10.8	4.7	6.7*	6	22	27	49	-	-	-	1.02	1.40	1.01	4
**XIY-026-R1**	A	17.6*	15.4	5.6	9.3*	5.1	27	25	52	94	-	-	1.14	0.98	2.85	4
**XIY-026-L1**	A	20.7	15	10	16.5	15	9	15	24	94	-	-	1.38	1.80	2.08	4
**XIY-027**	A	11.7	7.9	2.2	5.3	2.6*	24	25	49	-	-	-	1.48	2.00	1.70	4

**XIY-028-L1**	A	8.4*	7.2	-	7.2*	-	22	27	49	-	-	-	1.17	-	-	4
**XIY-028-R1**	A	10.5*	9.2	5.1	6.5	5.9	28	15	43	43	-	176	1.14	-	-	4
**XIY-028-L2**	A	9.8*	8.3	4.2	5.7*	5.3	24	20	44	41	84	175	1.18	1.58	0.98	4
**XIY-028-R2**	A	11.7	9	5	6	4.5	22	28	50	42	85	-	1.30	1.34	2.18	4
**XIY-029**	A	9.2*	7.7	-	3.4*	4.4	20	30	50	-	-	-	1.19	-	-	4
**XIY-031**	A	11.1*	9.6	4.6	7.8	5.2	44	44	88	-	-	-	1.16	1.75	3.72	4
**XIY-032**	A	15.7	9.8	5.7	11.3	6.3	18	18	36	-	-	-	1.60	1.41	1.52	4
**XIY-035**	A	20.5	11.3	11.2	15.1		35	-	-	-	-	-	1.81	-	-	4
**XIY-036**	A	13.7	7.7	6.4	11.4	6.7	11	13	24	-	-	-	1.78	-	1.19	4
**XIY-037**	A	19.4	15.3	7.4	11.3	11.8	23	19	42	-	-	-	1.27	-	-	4
**XIY-038**	A	14.5*	11.8	6.7	11.7	6.3	23	19	42	-	-	-	1.23	1.40	1.55	4
**XIY-039**	A	19.6*	-	12	15.4*	-	30	-	-	-	-	-	-	-	-	4
**XIY-040**	A	20.5	15	8.3	13.2	9.2	20	17	37	-	-	-	1.37	2.20	1.64	4
**XIY-041**	A	14.7*	14.7	6.6	12.2	8	30	20	50	-	-	-	1.00	-	-	4
**XIY-042-R1**	A	15.6*	14.6	8.6	9.4*	8.3*	16	22	38	46	-	-	1.07	-	-	4
**XIY-042-L1**	A	16.5	11.2	9.4	13.6	10.9*	15	14	29	-	-	-	1.47	1.29	1.46	4
**XIY-043**	A	10*	-	4.5*	7*	-	30	31	61	-	-	-	-	-	-	4
**XIY-044**	A	16	12	7.5	11.5	8	17	19	36	-	-	-	1.33	-	-	4
**XIY-047-R1**	A	19.2	17.2*	8.7	12.2	9.7*	39	25	64	58	-	-	1.12	-	2.69	3
**XIY-047-L1**	A	19.2*	18.7	6.2	10.4	9.9	32	33	65	-	-	-	1.03	1.23	3.45	3
**XIY-049**	A	10.4	6.6	4.9	7.4	5.2*	17	18	35	-	-	-	1.58	1.26	1.48	3
**XIY-050**	A	14	13	5.8	8.7	5.7	24	21	45	-	-	-	1.0	1.6	2.0	3

													8	0	8	
**XIY-051**	A	12.7*	11.8	4.3	6.6	4.6	21	26	47	-	-	-	1.08	1.54	1.61	3
**XIY-052**	A	16.4	15	9	12.3	12.4	48	26	74	-	-	-	1.09	1.00	1.76	2
**XIY-053**	A	15.5	12.7	7.5	10.5	8.8	20	40	60	-	-	-	1.22	1.50	2.01	3
**XIY-054**	A	14.6	10.8	5.6	8.5	5.8	27	20	47	-	-	-	1.35	2.65	2.80	2
**XIY-055**	A	17.9*	-	-	-	-	-	-	-	-	-	-	-	-	-	2
**XIY-056**	A	11.9*	9.5	9	7.7*	12.5	21	21	42	-	-	-	1.25	1.90	2.10	2
**XIY-057**	A	15*	13	8.6	11.4	8.6	16	16	32	-	-	-	1.15	-	-	2
**XIY-058**	A	16.6	13	6.2	9.6	7	18	34	52	-	-	-	1.28	1.63	1.75	2
**XIY-059**	A	15.6	13.2	5	10.9	6	28	24	52	-	-	-	1.18	1.64	2.39	2
**XIY-060-L1**	A	11*	9.6	4.6	7.8	6	46	20	66	44	-	-	1.15	0.85	1.12	2
**XIY-060-R1**	A	11.5*	11	4.4	10	6.6	34	77	111	-	-	-	1.05	1.25	2.48	2
**XIY-061**	A	9.6*	11.36	4.2	7.4*	5.8	27	43	70	-	-	-	0.85	1.40	2.14	2
**XIY-062**	A	13.6*	13.2	7.6	10.6*	6.3	35	35	70	-	-	-	1.03	2.40	2.22	2
**XIY-063**	A	14.8*	13	5.3	9.5	7	37	33	70	-	-	-	1.14	1.51	3.82	2
**XIY-064**	A	14.3*	15	5.9	-	5.7	49	45	94	-	-	-	0.95	-	-	2
**XIY-065**	A	18	15	8	14.3	9.4	18	23	41	-	-	-	1.20	1.33	1.38	2
**XIY-072**	A	13.6	9.4	4.8	7.1	3.9*	28	20	48	-	-	-	1.45	1.47	1.30	2
**XIY-073**	A	13.1	9.9*	4.1*	9.4	3.4*	39	23	62	-	-	-	1.32	-	-	2
**XIY-077**	A	15.8*	13.4	7.6	10	9.6	16	23	39	-	-	-	1.18	1.67	1.94	2
**XIY-078**	A	15.8*	16	7	10.2*	13.4	21	29	50	-	-	-	0.99	-	-	2
**XIY-079**	A	13*	11	5.6	7.6	6.6	20	18	38	-	-	-	1.1	1.1	1.3	2

													8	7	1	
**XIY-080**	A	13.2*	11	5.2	9.6	7.2	22	24	46	-	-	-	1.20	1.23	1.46	2
**XIY-081**	A	12.1	9.6	5	9	5.6	27	31	58	-	-	-	1.26	1.52	1.90	2
**XIY-085**	A	15.2*	11.7*	4.4	10.9	6.6*	40	37	77	-	-	-	1.3	-	-	2
**XIY-086**	A	16.8	13	5.8	10.5	8.2	37	22	59	-	-	-	1.29	1.51	1.86	2
**XIY-088**	A	16.8	14.2	7.1	12.7	8.3	17	16	33	-	-	-	1.18	1.84	1.55	2
**XIY-095**	A	-	8.4*	3.8	4.3	3.6*	34	31	65	-	-	-	-	-	-	2
**XIY-096**	A	12.9	9.1	6.7	9.6	7.4	16	17	33	-	-	-	1.42	1.65	1.48	2
**XIY-097**	A	17.8	11.5	8.4	9.8	9.2*	25	21	46	-	-	-	1.55	1.82	2.58	2
**XIY-098**	A	14.4*	-	-	8.8	1.8*	-	23	-	-	-	-	-	-	-	2
**XIY-101**	A	19	9	6.3	10.9	7.6	19	16	35	-	-	-	2.11	2.16	1.80	2
**XIY-102**	A	15*	13	6.3	9.6	7.6	22	24	46	-	-	-	1.15	2.00	2.07	2
**XIY-105**	A	18*	15.6	4.6	10	7.8	41	31	72	-	-	-	1.15	1.57	2.41	1
**XIY-106-L1**	A	13	7.7*	-	9.6	9.3	30	21	51	42	-	-	1.69	-	-	1
**XIY-106-R1**	A	14	12	5.6	11.3	7.3	42	32	74	-	-	-	1.17	1.50	1.40	1
**XIY-107**	A	14.3*	12.7	7.3*	10*	10.5*	31	15	46	-	-	-	1.13	1.27	1.60	2
**XIY-109**	A	15	14	8	9	8*	17	11	28	-	-	-	1.07	-	1.12	4
**XIY-110**	A	15	14	8	10	9	15	30	45	-	-	-	1.07	-	-	4
**XIY-111**	A	14.5*	13	8.5	11	8.5*	19	24	43	-	-	-	1.12	-	-	4
**XIY-112**	A	15.5	11	6.5	10.5	7.5	15	17	32	-	-	-	1.41	1.38	1.41	4
**XIY-070**	B	27	-	-	18	16	-	32	-	-	-	-	-	-	-	2
**XIY-071**	B	30	26	15	20	18	39	31	70	-	-	-	1.15	2.30	4.59	2

**XIY-074**	B	27.5	25.8	13	18	15.5	31	38	69	-	-	-	1.07	2.30	4.57	2
**XIY-075**	B	28	24*	-	24.6	20.7	45	25	70	-	-	-	-	-	-	2
**XIY-076**	B	-	-	14.2*	-	-	33	-	-	-	-	-	-	-	-	2
**XIY-087**	B	28.2*	23.4*	10.4*	18.8	15.2	40	22	62	-	-	-	1.21	-	-	2
**XIY-108**	B	27.4*	25	17	17	10	44	25	69	-	-	-	1.10	1.82	3.62	4
**XIY-048**	C	38.8	40.4	17	24.5	23	34	38	74	-	-	-	0.96	1.60	3.12	3

Table S2. Preservation scale for the tridactyl tracks from the Xiyang track site. Grades range from 0 to 2.5, following Marchetti et al. (2019). *: incomplete track.

Track number	Grade
**NO MORPHOTYPE**	
XIY-002	0.5
XIY-003	0
XIY-005	1
XIY-006	1
XIY-009	1
XIY-011	1
XIY-012	1
XIY-016	0.5
XIY-019	0.5
XIY-021	0
XIY-023	0.5
XIY-024	0.5
XIY-025	0
XIY-030	0.5
XIY-033*	0.5
XIY-034*	0.5
XIY-045*	0
XIY-046*	0
XIY-066*	0.5
XIY-067*	0.5
XIY-068*	0
XIY-069*	0.5
XIY-082*	0.5
XIY-083*	0.5
XIY-084*	0.5
XIY-089*	1
XIY-090*	1
XIY-091*	0.5
XIY-092*	0
XIY-093*	1
XIY-094*	1
XIY-099*	1
XIY-100*	1
XIY-103*	1
XIY-104*	1

MORPHOTYPE A	
XIY-001	1.5
XIY-004	0.5
XIY-007	1.5
XIY-008	1
XIY-010	1.5
XIY-013	1.5
XIY-014	2
XIY-015*	1.5
XIY-017*	1
XIY-018*	1
XIY-020	1
XIY-022	1
XIY-026-L1	1.5
XIY-026-R1	1.5
XIY-027	1.5
XIY-028-L1	1.5
XIY-028-R1	1.5
XIY-028-L2	1.5
XIY-028-R2	1.5
XIY-029	1.5
XIY-031	1.5
XIY-032	2
XIY-035	2
XIY-036	2
XIY-037	1
XIY-038	1.5
XIY-039*	1
XIY-040	1.5
XIY-041	1.5
XIY-042-L1	1
XIY-042-R1	1
XIY-043*	1.5
XIY-044	1.5
XIY-047-L1	1.5
XIY-047-R1	1.5
XIY-049	2
XIY-050	1.5
XIY-051	1.5
XIY-052	1.5
XIY-053	2.5

XIY-054	2
XIY-055*	1.5
XIY-056	1.5
XIY-057	1.5
XIY-058	1.5
XIY-059	1.5
XIY-060-L1	1
XIY-060-R1	1
XIY-061	1
XIY-062	1
XIY-063	1.5
XIY-064	1
XIY-065	2
XIY-072	1.5
XIY-073	1.5
XIY-077	1
XIY-078	1
XIY-079	1.5
XIY-080	1.5
XIY-081	2
XIY-085	1.5
XIY-086	2
XIY-088	2
XIY-095*	1.5
XIY-096	1.5
XIY-097	2
XIY-098	1.5
XIY-101	2
XIY-102	1.5
XIY-105	1.5
XIY-106-L1	1.5
XIY-106-R1	1.5
XIY-107	1
XIY-109	2
XIY-110	1.5
XIY-111	1
XIY-112	2
**MORPHOTYPE B**	
XIY-070*	1
XIY-071	2
XIY-074	1.5

XIY-075*	1.5
XIY-076*	0.5
XIY-087	1
XIY-108	2
**MORPHOTYPE C**	
XIY-048	2

Table S3. Specimen numbers and measurements of theropod ichnospecies holotypes from the Jurassic of China (except *Therangospodus* and *Gigandipus*, which are the paratypes). Abbreviations following the order of the table: L: maximum length (cm); W: maximum width (cm); PR: projection ratio following Li, 2015 (p. 9); CPR: "corrected" projection ratio following Olsen, Smith & McDonald, 1998 (p. 586).

Ichnospecies	Specimen number	L	W	PR	CPR
Changpeipus					
C. carbonicus	IVPP2472	40	24	1.91	2.87
C. longweimaoensis	NWUV 1407	25.4	18	1.92	3.01
Eubrontes					
E. monax	BPV-FP2	27.5	19	1.76	2.13
E. platypus	BPV-FP5	27	24	2.23	2.86
E. xiyangensis	BPV-FP6	26.5	17.5	2.15	2.48
E. zigongensis	ZDM 0032	42.1	30.5	1.80	2.51
E. glenrosensis	24-1	23	20	2.15	2.90
E. nianpanshanensis	CFZJ6	46	34	3.59	7.78
Gigandipus					
G. hei	CFZW46	49	37	3.08	3.21
Grallator					
G. limnosus	BPV-FP1	27.5	18.5	1.37	1.46
G. microiscus	CFZW176	14.5	8.5	1.64	1.71
G. wuhuangensis	CFNY8	17.8	12	1.54	2.73
G. yemiaoxiensis	CFZW101	9.8	5.5	1.45	1.81
Jialingpus					
J. yuechiensis	BMNH-Ph000467	20	12	1.35	1.98
Kayentapus					
K. xiaohebaensis	BPV-FP4	29	25	2.20	5.01
K. jizhaoshiensis	CFZW164	38.5	28	1.77	3.85
K. hailiutuensis	4-4	23	24	1.30	2.71
K. wumaensis	CFZW19	30	36.5	1.50	2.19
K. nananensis	CFNy1	30	25	2.30	4.12
Lufengopus					
L. dongi	LDRC028	40	35	3.40	10.33
Shensipus					
S. tuchuanghensis	NWUV 1416	9.4	8.7	1.47	7.25
Therangospodus					
T. pandemicus	CU-MWC 186.2	28	20	2.20	2.91

		Zhengichnus			
Z. jinningensis	BPV-FP7	28	19	0.40	0.92
		Zizhoupus			
Z. wangi	NWUV 1404	40	36.8	1.85	3.61

Table S4. Comparison of morphotype A with theropod ichnospecies reported from the Fengjiahe Formation. Abbreviations following the order of the table: L: maximum length; L/W: length over width ratio; div.: divarication angle; PR: projection ratio following Li, 2015 (p. 9). The main differences with morphotype A are in color.

	Average L	Average L/W	Average div. II^IV	Average PR
**Xiyang Morphotype A**	**14.4 cm**	**1.24**	**50°**	**1.6**
Eubrontes platypus	26.7 cm	1.12	34°	2.2
Eubrontes monax (ex *Paracoelurosaurichnus monax*)	27.5 cm	1.45	10°	1.8
Eubrontes xiyangensis (ex *Youngichnus xiyangensis*)	26.5 cm	1.51	21°	2.1
Grallator limnosus	< 15 cm	> 2.00	35°	1.4
Kayentapus xiaohebaensis (ex *Schizograllator xiaohebaensis*)	29 cm	1.16	75°	2.2
Zhengichnus jinningensis	28 cm	1.47	100°	0.4

Table S5. Calculated hip heights and body lengths for the 3 morphotypes. Values presented for morphotypes A and B are averages. Hip height is calculated following two different approaches (see material & methods).

	Morphotype	Hip height	Body length
**Thulborn, 1990**	A	0.7 m	2.6 m
	B	1.4 m	5.5 m
	C	1.9 m	7.3 m
**Alexander, 1976**	A	0.6 m	2.3 m
	B	1.4 m	4.5 m
	C	1.6 m	6.6 m

References

Alexander RM. 1976. Estimates of speeds of dinosaurs. Nature 261:129-130 DOI: 10.1038/261129a0.

Li JJ. 2015. *Palaeovertebrata Sinica Volume II Footprints of Mesozoic Reptilians and Avians Fascicle 8*. Beijing: Science Press.

Marchetti L, Belvedere M, Voigt S, Klein H, Castanera D, Diaz-Martinez I, Marty D, Xing L, Feola S, Melchor RN, Farlow JO. 2019. Defining the morphological quality of fossil footprints. Problems and principles of preservation in tetrapod ichnology with examples from the Palaeozoic to the present. *Earth Science Reviews* 193:109-145.

Olsen PE, Smith JH, McDonald NG. 1998. Type material of the type species of the classic theropod footprint genera *Eubrontes*, *Anchisauripus* and *Grallator* early Jurassic, Hartford and Deerfield basins, Connecticut and Massachusetts, USA. *Journal of Vertebrate Paleontology* 18: 587-601.

Thulborn T. 1990. *Dinosaur Tracks*. London: Chapman and Hall.
